# Adapted Beamforming: A Robust and Flexible Approach for Removing Various Types of Artifacts from TMS–EEG Data

**DOI:** 10.1007/s10548-024-01044-4

**Published:** 2024-04-10

**Authors:** Johanna Metsomaa, Yufei Song, Tuomas P. Mutanen, Pedro C. Gordon, Ulf Ziemann, Christoph Zrenner, Julio C. Hernandez-Pavon

**Affiliations:** 1 Department of Neuroscience and Biomedical Engineering, Aalto University School of Science, P.O. Box 12200, FI-00076 AALTO Espoo, Finland; 2 Hertie-Insitute for Clinical Brain Research, University of Tübingen, Tübingen, Germany; 3 Department of Neurology and Stroke, University of Tübingen, Tübingen, Germany; 4 Centre for Addiction and Mental Health, Toronto, Canada; 5 Department of Psychiatry, University of Toronto, Toronto, Canada; 6 Institute for Biomedical Engineering, University of Toronto, Toronto, Canada; 7 Department of Psychological Sciences, Kansas State University, Manhattan, KS USA

**Keywords:** Artifacts, Beamforming, Electroencephalography, Transcranial magnetic stimulation

## Abstract

Electroencephalogram (EEG) recorded as response to transcranial magnetic stimulation (TMS) can be highly informative of cortical reactivity and connectivity. Reliable EEG interpretation requires artifact removal as the TMS-evoked EEG can contain high-amplitude artifacts. Several methods have been proposed to uncover clean neuronal EEG responses. In practice, determining which method to select for different types of artifacts is often difficult. Here, we used a unified data cleaning framework based on beamforming to improve the algorithm selection and adaptation to the recorded signals. Beamforming properties are well understood, so they can be used to yield customized methods for EEG cleaning based on prior knowledge of the artifacts and the data. The beamforming implementations also cover, but are not limited to, the popular TMS–EEG cleaning methods: independent component analysis (ICA), signal-space projection (SSP), signal-space-projection-source-informed-reconstruction method (SSP–SIR), the source-estimate-utilizing noise-discarding algorithm (SOUND), data-driven Wiener filter (DDWiener), and the multiple-source approach. In addition to these established methods, beamforming provides a flexible way to derive novel artifact suppression algorithms by considering the properties of the recorded data. With simulated and measured TMS–EEG data, we show how to adapt the beamforming-based cleaning to different data and artifact types, namely TMS-evoked muscle artifacts, ocular artifacts, TMS-related peripheral responses, and channel noise. Importantly, beamforming implementations are fast to execute: We demonstrate how the SOUND algorithm becomes orders of magnitudes faster via beamforming. Overall, the beamforming-based spatial filtering framework can greatly enhance the selection, adaptability, and speed of EEG artifact removal.

## Introduction

Transcranial magnetic stimulation combined with electroencephalography (TMS–EEG) is a safe and non-invasive technique that has been successfully used for basic and clinical research (Tremblay et al. 2019). TMS–EEG allows one to investigate cortical connectivity and the neurophysiology of the human brain (Ilmoniemi et al. 1997; Hernandez-Pavon et al. 2023). However, there are still challenges in the application of TMS–EEG (Hernandez-Pavon et al. 2023). A significant issue is that the transcranial magnetic stimulation-evoked EEG interpretation is complicated by the presence of high-amplitude muscle artifacts elicited by TMS depending on the brain area stimulated (Korhonen et al. 2011; Mutanen et al. 2013). In addition to muscle artifacts, many other artifacts of physiological and non-physiological nature can be elicited (Ilmoniemi et al. 2015; Rogasch et al. 2017; Varone et al. 2021; Hernandez-Pavon et al. 2022). More recently, the importance of effectively separating the peripheral-evoked potentials from those evoked by direct cortical stimulation, both due to TMS, has been brought up by multiple recent studies (Conde et al. 2019; Biabani et al. 2019; Gordon et al. 2021). Accurate removal of these artifacts and irrelevant data is a key step to make reliable interpretations of the neurophysiological recordings.

To recover the neuronal data, masked by the artifacts, a number of computational techniques have been developed, for an extensive review see Hernandez-Pavon et al. (2022). Independent component analysis (ICA), is perhaps the most popular method (Korhonen et al. 2011; Hernandez-Pavon et al. 2012; Rogasch et al. 2014; Wu et al. 2018). Signal-space projection (SSP) is another widely applied method, where one first needs to identify a set of topographies spanning the artifact subspace, and then a projection operation can be used to delete this subspace out of the EEG data (Uusitalo and Ilmoniemi 1997; Mäki and Ilmoniemi 2011). While the projection can eliminate some neuronal data, this loss can be taken into account by the EEG source analysis, an idea that was used in Mutanen et al. (2016) to introduce the signal-space-projection–source-informed-reconstruction method (SSP–SIR). Somewhat related to SSP–SIR, the source-estimate-utilizing noise-discarding algorithm (SOUND) was later developed to suppress EEG sensor noise (Mutanen et al. 2018). A less utilized technique is the multiple-source approach, where we define the multi-sensor distributions (topographies) of all underlying signal generators, neural and artifactual, a priori, after which we can decompose and separate the artifact data using the method of least squares (Berg and Scherg 1994; Litvak et al. 2007).

Studies comparing the TMS–EEG cleaning techniques show that the outcome significantly depends on the algorithms used (Bertazzoli et al. 2021; Rogasch et al. 2022), which raises the question of which results are reliable. Unfortunately, we lack the ground truth signals for measured TMS–EEG data, which makes it very difficult to conclude the recommended algorithms. It is poorly understood what lies behind the differing outcomes. There is also no good consensus or reasoning about when and why one should use which cleaning method (Hernandez-Pavon et al. 2022, 2023).

While establishing ground truth data, e.g., via simulations, can be highly useful for comparisons, we also believe that taking better into account the assumptions and properties of the different methods can facilitate the design of the optimal cleaning workflows for given data sets. Instead of describing the methods as completely separate entities, we should rather view them as branches of the same basic idea of linear spatial filtering. By writing these methods in terms of the same spatial-filtering framework, allows us to conclude where the observed differences are actually arising from Hernandez-Pavon et al. (2022).

Indeed, the spatial-filtering-based cleaning methods listed above can be brought under the same theoretical framework of beamforming (BF) as was shown in Hernandez-Pavon et al. (2022). Thus, these methods can be viewed as a continuum of the same basic idea. Importantly, as the assumptions and properties of beamforming are well known (Van Veen et al. 1997; Hui and Leahy 2006), we are able to make direct comparisons across the methods and to deduce the differences in the outcomes. For example, we know that beamforming for a single source (artifactual or neural) is able to retrieve the part of the time course, which is uncorrelated from all the other time-courses underlying the data (Van Veen and Buckley 1988; Van Veen et al. 1997; Hernandez-Pavon et al. 2022), so we can formulate the single-sensor noise elimination using beamforming.

Because beamforming formulation is simple, and its theory and properties are well understood, an endless number of beamforming-based spatial filters can be derived in a straightforward manner. One can tailor the cleaning to make it best-compatible with different types of artifacts, data and research questions. Here, we overview the basic principle of how to use the beamforming method for artifact elimination, and the choices that can be made in designing the BF filter. We then illustrate various practical applications for this framework.

To quantitatively test the beamforming-based EEG cleaning, we use measured and simulated data as well as multiple parameter selections to compute the BF filter to extensively cover all the basic types of usage. Our approach of mixing measured data and simulated artifact provides a way to measure how well the artifact rejection works as we know the ground truth data. As we can measure the accuracy of the data cleaning, our results allow us to make practical recommendations for the optimal usage of spatial filtering in different conditions.

When implementing beamforming-based artifact or noise removal, one has to set a few modelling variables and parameters based on the assumed artifact and data properties. These choices are taken into account by the beamforming formula, which retrieves us the final filtering algorithm. In this manner, novel adapted data-cleaning methods are rather straightforward to design and compute. We show with measured data, how different types of artifacts and noise/artifact signals can be filtered out with such an approach. Moreover, we illustrate how peripheral-evoked parts of TMS-evoked potentials (TEPs) can be erased from true TMS-evoked data making use of beamforming informed by sham-evoked EEG. BF is also easily adapted to cases when the data statistics or the topographies are modified, as we illustrated with ocular artifacts.

In addition to simplifying the cleaning by spatial filtering, beamforming can also greatly speed up the computation as the implementation is simplified. Here, this is demonstrated for SOUND, as it turns out that the novel formulation reduces the number of computationally expensive inverse operations by the number of the EEG channels. This is advantageous especially for online EEG cleaning, where short run-time is a crucial feature for the chosen algorithm. Additionally, to optimally de-noise non-stationary data, multiple data segments would benefit from separately computed cleaning filters to adapt to the changes in the data. However, such application can prove impractical unless the algorithm is highly efficient in speed.

## Methods

### Background Theory

#### Beamforming-Based Data Cleaning

The theory behind the linear filtering of TEPs as well as the modelling of TMS–EEG data has been extensively covered in Hernandez-Pavon et al. (2022). Here, we briefly outline the fundamental mathematical formulations required for beamforming-based data cleaning.

We assume that the recorded $M_{\mathrm {}}\times T$ data matrix $\textbf{X}$, from an $M_\mathrm {}$-channel EEG recording containing *T* samples, are due to *N* hidden components, of which $N_\textrm{brain}$ are of neuronal and $N_\textrm{art}$ of artifactual origin. The data are generated by the summation of the neural signals $\textbf{X}_\textrm{brain}$ and artifactual data $\textbf{X}_\textrm{art}$, which arise according to the linear model:

1 $$\begin{aligned} \textbf{X}=\textbf{X}_\textrm{brain}+\textbf{X}_\textrm{art}=\textbf{A}_\textrm{brain}\textbf{S}_\textrm{brain}+\textbf{A}_\textrm{art}\textbf{S}_\textrm{art}=\begin{bmatrix} \textbf{A}_\textrm{brain},\; \textbf{A}_\textrm{art} \end{bmatrix}\begin{bmatrix} \textbf{S}_\textrm{brain} \\ \textbf{S}_\textrm{art} \end{bmatrix}=\textbf{AS}, \end{aligned}$$

where $\textbf{A}_\textrm{brain}$, $\textbf{A}_\textrm{art}$, and $\textbf{S}_\textrm{brain}$, $\textbf{S}_\textrm{brain}$ are the mixing matrices and the time-course matrices for neuronal, and artifactual components generating the data, respectively. Concatenating the neuronal and artifactual mixing matrices horizontally and the time-courses vertically yields the total $M_\mathrm {}\times {N}$ mixing and the ${N} \times T$ time-course matrices $\textbf{A}$, and $\textbf{S}$, respectively. The columns of the mixing matrix are the topographies and the rows of the time-course matrices are the waveforms of the components, denoted by $\textbf{s}_i^\mathrm T$ for component *i*. Often times, a noise data matrix $\textbf{N}$ with the same dimensions as the data is added as a separate term in Eq. (1), i.e., $\textbf{X}=\textbf{AS}+\textbf{X}_\textrm{noise}$. Here, we consider noise to be generated by underlying noise components $\textbf{X}_\textrm{noise}=\textbf{A}_\textrm{noise}\textbf{S}_\textrm{noise}$. Throughout this paper, we assume that the temporal activity of the neural, artifactual, and noise components are mutually uncorrelated and that their topographies are not affected by the activity of the other components. Additional assumptions may be set, and defined separately in conjunction with each specific type of data cleaning application.

We now consider a spatial filter $\textbf{w}_i$ that uncovers the time-course of source *i* as $\textbf{s}_i^\mathrm T= \textbf{w}_i^\mathrm T \textbf{X}$. To extract the time-courses of all the artifact components, we would need a spatial filter matrix $\textbf{W}_\textrm{art}$, with all the spatial filter vectors in the columns, to be applied as $\textbf{W}_\textrm{art}^\mathrm T\textbf{X}=\textbf{S}_\textrm{art}$. The respective artifact data are given as

2 $$\begin{aligned} \textbf{X}_\textrm{art}=\textbf{A}_\textrm{art}\textbf{S}_\textrm{art}=\textbf{A}_\textrm{art}\textbf{W}_\textrm{art}^\textrm{T}\textbf{X}. \end{aligned}$$

Popular TMS–EEG data cleaning methods, ICA, SSP(–SIR), SOUND, and the Berg–Scherg methods, are based on spatial filtering, which can be expressed as matrix multiplications from the left by the cleaning matrix $\textbf{M}_{\textrm{clean}}$:

3 $$\begin{aligned} \textbf{M}_{\textrm{clean}}\textbf{X}=\textbf{M}_{\textrm{clean}}\textbf{AS}=[\textbf{M}_{\textrm{clean}}\textbf{A}_\textrm{brain},\; \textbf{M}_{\textrm{clean}}\textbf{A}_\textrm{art}]\begin{bmatrix} \textbf{S}_\textrm{brain} \\ \textbf{S}_\textrm{art} \end{bmatrix}\approx [\textbf{A}_\textrm{brain},\; \textbf{0}]\begin{bmatrix} \textbf{S}_\textrm{brain} \\ \textbf{S}_\textrm{art} \end{bmatrix}=\textbf{A}_\textrm{brain}\textbf{S}_\textrm{brain}, \end{aligned}$$

where the artifact topographies are set close to zero, while the neuronal topographies would optimally stay intact. With the help of Eq. (2), we can construct the optimal cleaning matrix $\textbf{M}_{\textrm{clean}}$, to get purely neuronal data $\textbf{X}_\textrm{brain}$, as

4 $$\begin{aligned} \textbf{M}_{\textrm{clean}}\textbf{X}=(\textbf{I}-\textbf{A}_\textrm{art}\textbf{W}^\textrm{T}_\textrm{art})\textbf{X}=\textbf{AS}-\textbf{A}_\textrm{art}\textbf{S}_\textrm{art}=\textbf{A}_\textrm{brain}\textbf{S}_\textrm{brain}=\textbf{X}_\textrm{brain}. \end{aligned}$$

In Hernandez-Pavon et al. (2022), it was shown that we can formulate all existing spatial filter-based cleaning techniques within the framework of beamforming, by which the spatial filter matrix for Eq. (4) is obtained as

5 $$\begin{aligned} \textbf{W}^\mathrm {}_\textrm{art}=\Sigma _\mathrm {}^{-1} \textbf{A}_\textrm{art} (\textbf{A}_\textrm{art}^\mathrm T \Sigma _\mathrm {}^{-1} \textbf{A}_\textrm{art})^{-1}, \end{aligned}$$

where $\Sigma _\mathrm {}$ is the data covariance matrix. As neither the true artifact topographies nor the covariance matrix are known, we use their estimates $\hat{\Sigma }$, $\hat{\textbf{A}}_\textrm{art}$ instead. The advantage of Eq. (5) is that the single formula can be used to derive novel adapted EEG cleaning approaches for various artifacts and data. In practice, this is accomplished by estimating $\hat{\Sigma }$, and $\hat{\textbf{A}}_\textrm{art}$ in different ways as described later.

To estimate the beamforming filter by Eq. (5), we need to define the topographies spanning the artifact subspace, the data covariance matrix, and the regularization for computing the inverse of the covariance. There are two main options for estimating the data covariance matrix. In context of beamforming, sample-based covariance is most often used as $\Sigma _\mathrm {}\approx \textbf{XX}^\textrm{T}/T$. As discussed in (Hernandez-Pavon et al. 2022), the sample-based estimate can get biased with evoked (time-dependent) components, so mean-subtraction, suggested in (Metsomaa et al. 2016), is useful. Mean subtraction is simply obtained by subtracting the trial-averaged evoked response $\varvec{\bar{X}}$ from each single-trial response $\textbf{X}_r$ in trial *r*, which yield the sample-based covariance estimate as

6 $$\begin{aligned} \hat{\Sigma }^\textrm{sample}=\langle (\textbf{X}_r-\varvec{\bar{X}} )(\textbf{X}_r-\varvec{\bar{X}})^\textrm{T}\rangle _{r}\, \end{aligned}$$

where $\langle \cdot \rangle _r$ denotes taking sample mean over the trials.

Another approach is to use the model-based covariance matrix, which is estimated by

7 $$\begin{aligned} \hat{\Sigma }^\textrm{model}=\Sigma _{\textrm{brain}}+\Sigma _{\textrm{art}}+\Sigma _{\textrm{noise}}=\textbf{A}_{\textrm{brain}}\Lambda _{\textrm{brain}}\textbf{A}_{\textrm{brain}}^\textrm{T}+\textbf{A}_{\textrm{art}}\Lambda _{\textrm{art}}\textbf{A}_{\textrm{art}}^\textrm{T}+\textbf{A}_{\textrm{noise}}\Lambda _{\textrm{noise}}\textbf{A}_{\textrm{noise}}^\textrm{T}, \end{aligned}$$

where $\Sigma _{\textrm{brain}}$, $\Sigma _{\textrm{art}}$, and $\Sigma _{\textrm{noise}}$ are the data covariance matrices of the three types of data, neuronal, artifactual, and noise, respectively, while $\Lambda _{\textrm{brain}}$, $\Lambda _{\textrm{art}}$, and $\Lambda _{\textrm{noise}}$ are the covariance matrices of the corresponding underlying components. The covariance matrices of the three data types add up since they are assumed mutually uncorrelated. We note here, that strictly speaking, temporally coexisting artifacts and neural activity may appear correlated; few preprocessing steps, including baseline resetting or the so-called ’mean-subtraction’, have been previously proposed to overcome this issue (Metsomaa et al. 2014, 2016; Hernandez-Pavon et al. 2022).

To use Eq. (7), we should define the mixing matrices and the component covariance matrices. Common choices for the covariance matrices are diagonal matrices, e.g., $\Lambda =\textrm{diag}(\lambda _1, \ldots , \lambda _N)$. In the simplest case, all diagonals are set uniform $\Lambda =\textbf{I}\lambda$. Similarly to the source localization problem, the neuronal mixing matrix $\textbf{A}_\textrm{brain}$ can be computed by, e.g., boundary-element model after inserting a set of distributed dipolar sources in a multi-compartment head model, yielding the so-called lead-fied matrix.

The topographies of the artifacts for using Eqs. (4), (5), and (7) could be estimated using statistical methods, for example, ICA or principal component analysis (PCA). In “Estimating Artifact Topographies” section, some more detailed suggestions are given for topographic estimation. Different ways of choosing the artifact topographies and covariance matrices yield different previously published cleaning algorithms as has been shown in Hernandez-Pavon et al. (2022).

Importantly, we are not limited to the established spatial-filter -based cleaning methods, but we may use Eq. (5) creatively to optimize the cleaning result for the data at hand. For example, the covariance matrix could be estimated using the sample- or model-based estimation, or their combination. In the following sections, we illustrate several types of implementations based on Eqs. (4) and (5) to accommodate the needs of different types of data and artifacts.

#### Estimating Artifact Topographies

Perhaps the most challenging part of preparing the spatial filter operator $\textbf{W}_\textrm{art}$ by Eq. (5) is to define a set of topographies representing the artifact components $\textbf{A}_\textrm{art}$. While for the neuronal EEG data, we can use physical modelling to estimate the mixing matrix $\textbf{A}_\textrm{brain}$, for the artifact components such a model is commonly not available. Thus, we need to make use of the statistical properties of the data to estimate topographies which most likely represent artifacts. Here, we describe different ways to estimate the artifact topographies to get $\hat{\textbf{A}}_\textrm{art}$, which can then be inserted into (5) to derive the spatial filter.

Importantly, two properties of beamforming make the artifact topography estimation task easier. Firstly, we do not need to define the topography $\textbf{a}_{\textrm{art},i}$ of each artifact component *i*. Instead, we can estimate a set of $\hat{N}$ topographies ${\hat{\textbf A}}_\textrm{art}=[{\hat{\textbf a}}_{\textrm{art},1},\;\ldots ,\;{\hat{\textbf a}}_{\textrm{art},\hat{N}}]$, which together span the artifact subspace, i.e., all true artifact topographies can be represented as a weighted sums of the estimated topographies, $\textbf{a}_{\textrm{art},i}=\sum _j c_{i,j} \,{\hat{\textbf a}}_{\textrm{art},j}$, where $c_{i,j}$’s denote the weights. Moreover, the span of the estimated topographies does not need to be exactly same as the span of the true artifacts; it is enough if the estimated span includes the true span:

8 $$\begin{aligned} \textrm{span}(\textbf{A}_\textrm{art})\subset \textrm{span}({\hat{\textbf A}}_\textrm{art}), \end{aligned}$$

which means that the estimate artifact subspace may also contain some part of artifact-free data (Hernandez-Pavon et al. 2022). However, the wider the span of the estimated artifact subspace, the greater the risk for undesired suppression of neuronal EEG signals. If the span fully includes some neuronal EEG topographies, the cleaning will also remove these interesting signals completely because beamforming by (5) yields the time course estimates ${\hat{\textbf S}}_\textrm{art}$ for all components lying within the span of ${\hat{\textbf A}}_\textrm{art}$; for detailed theoretical reasoning please see Hernandez-Pavon et al. (2022). Such biased cleaning is termed as overcorrection. The opposite effect, undercorrection, takes place if some artifact topography is partly not included in the span of the estimated artifact subspace. We also note here that, alternatively, one could as well determine the topographies spanning the neural EEG $\textbf{A}_\textrm{brain}$, and then use beamforming to extract the clean data for such a neural subspace. In this case, the practical challenge are the leakage signals from high-amplitude artifacts into neural EEG estimates because, in our experience, it is difficult to extract a sufficiently accurate set of neural basis topographies (by PCA or other means).

In general, the most common ways to estimate artifact topographies are PCA and ICA. As ICA assumes statistical independence, one should carefully think whether this assumption is valid for the types of artifacts that are removed. If the assumption is invalid, the ICA-estimated topographies may combine all EEG activity overlapping temporally with the artifact (Hernandez-Pavon et al. 2022; Metsomaa et al. 2014).

PCA is useful in extracting the topographies, which cover most of the data power. Prior to PCA, it can be beneficial to apply temporal filtering to the data. This $T\times T'$ filter $\textbf{F}$, retrieving a filtered waveforms of length $T'$, should be designed so as to enhance the artifact-to-signal-ratio of the EEG since the topographies stay intact in temporal filtering applied as $\textbf{X} \textbf{F}=\textbf{AS} \textbf{F}=\textbf{A}\tilde{\textbf{S}}$. This idea was introduced in Mäki and Ilmoniemi (2011), where high-pass filtering was applied to highlight the TMS-evoked muscle artifacts which also contain power within high frequencies.

We would like to emphasize that different types of temporal filters can be useful before PCA, depending on the properties of the data and artifacts. For example, a simple $T\times T-1$ difference filter $\textbf{F}^\textrm{diff}$, defined as

9 $$\begin{aligned} \begin{aligned} {F}^\textrm{diff}_{i,j}&=1,\,\,\,\,\,\,\textrm{when}\, i=j \\ {F}^\textrm{diff}_{i,j}&=-1,\, \textrm{when}\, i=j+1\\ F^\textrm{diff}_{i,j}&=0,\, \,\,\,\,\,\textrm{otherwise}, \end{aligned} \end{aligned}$$

can be used to highlight rapidly and monotonically changing artifact components. If there is a spike-like artifact, meaning a short-lived high-amplitude deflection, a $T\times T-2$ second-order difference filter (Laplacian) $\textbf{F}^\textrm{Lap}$ may be beneficial:

10 $$\begin{aligned} \begin{aligned} {F}^\textrm{Lap}_{i,j}&=1,\, \,\,\,\,\,\textrm{when}\, i=j \\ {F}^\textrm{Lap}_{i,j}&=-2,\, \textrm{when}\, i=j+1\\ F^\textrm{Lap}_{i,j}&=1,\, \,\,\,\,\,\textrm{when}\, i=j+2\\ F^\textrm{Lap}_{i,j}&=0,\, \,\,\,\,\,\textrm{otherwise}. \end{aligned} \end{aligned}$$

The goal of the temporal filtering is to ensure that the artifacts have a high relative power compared to other signals. PCA then is run and a set of topographies containing most of the power are set as the columns of ${\hat{\textbf A}_\textrm{art}}$ to estimate the spatial span of the artifacts.

In some cases, the artifact topographies are set based on physical generative assumptions. In SOUND and in the data-driven Wiener filter (DDWiener) (Mutanen et al. 2018), we assume that the artifact/noise topographies show non-zero activity in one electrode only. Thus, we can set all such single-sensor topographies as separate uncorrelated artifacts. See “Case 2: Implementation of Fast SOUND via Beamforming” section, for a detailed description of how to formulate SOUND via beamforming.

It may also be possible to measure artifact EEG $\textbf{X}_\textrm{art}$ purely generated by the problematic sources. By directly applying PCA to these artifact data, we can obtain the basis for artifact topographies. For instance, TMS-evoked EEG also contain auditory- and somatosensory-evoked neural responses, which are difficult to separate from the direct cortical-evoked TEPs. Sham-stimulation, only delivering the peripheral stimuli, has been suggested as a control condition for TMS. The sham-evoked data are driven by non-interesting mechanisms only, so we may treat them as artifact data $\textbf{X}_\textrm{art}$. Such control-condition recording is described in detail in “Experiment 2: Realistic Sham TMS Versus Real TMS at Supplementary Motor Area” section, and in “Adaptive Cleaning with Non-stationary Data Covariance or Changing Artifact Patterns” section, where we explain how to remove the peripheral-evoked potentials from the TEPs by beamforming.

#### Different Regularization Types

The usage of beamforming (Eq. (5)) requires inverting the data covariance matrix. In practice, the covariance matrix is often ill-posed since the number of channels exceeds the number of sufficiently high-amplitude components in the data (degrees of freedom). The ill-posedness results in unstable beamforming filters due to the numerical problems in the inversion, for which reason regularization is used. Here, we consider three types of regularization briefly described below.

When eigendecomposition (ED) is applied to the covariance matrix $\Sigma$, we get

11 $$\begin{aligned} \Sigma _\mathrm {}=\textbf{U D U}^\textrm{T}=\sum _{i=1}^{M_\mathrm {}} d_{i} \textbf{u}_i \textbf{u}_i^\textrm{T}, \end{aligned}$$

where $M_\mathrm {}\times M_\mathrm {}$ $\textbf{U}=[\textbf{u}_1,\,\ldots ,\, \textbf{u}_{M_\mathrm {}}]$ is an orthogonal matrix with eigen vectors as columns, and $M_\mathrm {}\times M_\mathrm {}$ $\textbf{D}$ is a diagonal matrix with eigenvalues, $d_1,\,\ldots ,\,d_{M_\mathrm {}}$, as its diagonal elements in a descending order. This eigendecomposition is also used for retrieving the components in PCA, where the eigenvectors are interpreted as the EEG (basis) topographies.

Inverse of the covariance matrix becomes

12 $$\begin{aligned} \Sigma _\mathrm {}^{-1}=\textbf{U D}^{-1} \textbf{U}^\textrm{T}=\sum _{i=1}^{M_\mathrm {}} d_{i}^{-1} \textbf{u}_i \textbf{u}_i^\textrm{T}. \end{aligned}$$

In the case of EEG, there are several eigenvalues which are close or equal to 0. Numerical problems arise when the small eigenvalues turn into very large inverted values, dominating the inverse matrix computation in Eq. (12).

To avoid the numerical problems, regularization is applied. Tikhonov regularization equals to setting $\Sigma \leftarrow \Sigma +\textbf{I}\gamma,\; \mathrm{with\; \gamma>0}$. As a result, the inverse becomes

13 $$\begin{aligned} \Sigma ^{\dagger ,\textrm{Tikhonov}}=\textbf{U}( \textbf{D}+ \gamma \textbf{I})^{-1} \textbf{U}^\textrm{T} =\sum _{i=1}^{M_\mathrm {}} (\gamma +d_{i})^{-1} \textbf{u}_i \textbf{u}_i^\textrm{T}, \end{aligned}$$

which reduces the contribution of the small eigenvalues in the inverse estimate.

The eigenvalue truncation-based regularization means that the small eigenvalues are cut out and only *P* largest values are preserved, giving

14 $$\begin{aligned} \Sigma ^{\dagger ,\textrm{ED}}=\sum _{i=1}^P d_{i}^{-1} \textbf{u}_i \textbf{u}_i^\textrm{T}. \end{aligned}$$

Note that this regularization is similar to the so called singular-value truncation technique, which is commonly used in EEG source estimation, when inverting the lead-field matrix. Additionally, preserving the largest components is also often the application for which PCA is used to reduce data dimensionality for simplifying the data interpretation/analysis.

The third type tested here is based on the alternative formulation of beamforming derived in Hernandez-Pavon et al. (2022). As a prerequisite, we need to have an orthonormal set of basis vectors spanning the estimated artifact subspace set as columns of ${\hat{\textbf A}_\textrm{art}}$, which fully spans the artifacts as defined in Eq. (8). We also need its orthocomplement, which only contains neuronal data and is spanned by another orthonormal set of topographies, the columns of $\textbf{B}$. In practice, this orthocomplement is retrieved from PCA as the topographies remaining after extracting the artifact topographies, and thus indexed by ${\hat{N},\hat{N}+1,\ldots , M}$, which are considered purely neural. Consequently, ${\hat{\textbf A}_\textrm{art}}$ and $\textbf{B}$ span the entire data. It is noteworthy that $\textbf{B}$ is not an estimate of $\textbf{A}_\textrm{brain}$ because the estimated artifact subspace may (and is allowed to) partly overlap with neural EEG as discussed in “Estimating Artifact Topographies” section.

Now the beamforming filter is retrieved by

15 $$\begin{aligned} \textbf{W}^\mathrm {}_\textrm{art}= {\hat{\textbf A}_\textrm{art}}-\textbf{B} (\textbf{B}^\mathrm T{\Sigma }\textbf{B})^{-1} \textbf{B}^\mathrm T \Sigma {\hat{\textbf A}_\textrm{art}} , \end{aligned}$$

where we use the eigenvalue truncation -based pseudoinverse (similar to Eq. (14)) to compute the inverse in the second term: $(\textbf{B}^\mathrm T{\Sigma }\textbf{B})^{\dagger ,ED}$. Taking a closer look at this invertable matrix, we see that it includes the EEG covariance matrix projected to the subspace defined by the columns of $\textbf{B}$, which is free of artifacts, so the inverse is applied to a covariance matrix of dimensions $(M-\hat{N}) \times (M-\hat{N})$. Here, we refer to this regularization as ED regularization type 2, while the conventional ED truncation by Eq. (14) is referred to as ED regularization type 1. We compare the two types of ED regularization with simulated data as described in “Simulating and Cleaning Artifactual TEPs” and “Simulation Results” sections.

#### Adaptive Cleaning with Non-stationary Data Covariance or Changing Artifact Patterns

Using the BF-based cleaning by Eqs. (5) and (4), we assume that the covariance matrix and the artifact topographies stay fixed. In some cases, however, it may be useful to adapt the cleaning matrix to the changes in the data covariance. For example, we might expect that the data statistics are changing as a function of time after a TMS pulse, as also illustrated by the fact that averaged TEPs consist of time-dependent deflections. Additionally, during a long measurement, the data statistics may be changing, e.g., the sensor noise levels (the noise variances) can change, which needs to be taken into account in real-time data cleaning (Makkonena et al. 2021). As BF makes use of the data covariance matrix, the changes in this variable should be taken into account, for example, by windowing the data into segments within which we can assume stationarity. Commonly, we can expect that a particular artifact preserves the same topographic pattern throughout a measurement session if the physical phenomenon generating the pattern stays the same. In such a case, we can simply update the cleaning matrix, by updating the data covariance matrix estimate in Eq. (5). For example, in real-time cleaning, we could expect that eye blinks topographies keep constant. Hence, it is enough to estimate them once, after which blink removal requires re-estimating the covariance, which idea was presented in Makkonena et al. (2021).

There may also be cases where the artifact topographies are changing significantly, so that the artifact mixing matrix should be adapted to the time window at hand. If TEPs contain several types of artifacts, which can be divided into separate windows, it can be feasible to use a separate subset of artifact topographies when applying Eqs. (5) and (4) in each window.

The artifact topographies may also change due to data processing. If the data in Eq. (1) are modified by multiplying by a spatial filter matrix $\textbf{P}_\textrm{spat}$, the underlying mixing matrix is modified accordingly. This modifies the artifact topographies as $\tilde{\textbf{A}}_\textrm{art}=\textbf{P}_\textrm{spat}\textbf{A}_\textrm{art}$, along with all the other topographies. Naturally, spatial filtering also modifies the covariance matrix by $\tilde{\Sigma }=\textbf{P}_\textrm{spat}\Sigma \textbf{P}_\textrm{spat}^\textrm{T}$. Inserting these new terms into Eq. (5) and assuming that $\textbf{P}_\textrm{spat}$ is invertible gives the modified spatial filter:

16 $$\begin{aligned} \tilde{\textbf{W}}_\textrm{art}= \textbf{P}_\textrm{spat}^{-\textrm{T}}{\textbf{W}}_\textrm{art}. \end{aligned}$$

In practice, computing the cleaning matrix starting from the definition in Eqs. (4) and (5) with appropriate regularization could be preferable over the shortcut version given in Eq. (16) because $\textbf{P}_\textrm{spat}$ are often not stable to invert as such, which may lead to distorted cleaning results. In “Case 4: Adaptive BF-Based Cleaning to Eliminate Peripheral-Evoked Potentials” section, we explain how to make use of adaptive cleaning approaches in removing ocular artifacts and peripheral-evoked responses from TEPs.

### Data Measurement and Preprocessing

Two types of data were recorded, which were used to demonstrate various BF-based cleaning approaches described in “Analysis of the Measurement Data” section. *We would like to point out that the used cleaning pipelines or selections for the parameters are not presented as the optimal ones for TEP preprocessing*. Other choices could also be well justified. Our aim is rather to demonstrate illustrative example cases on how to make use of the beamforming-based cleaning for various types of artifacts.

#### Participants

The study participants were healthy right-handed adults (female; 25 and 27 years). The participants gave their written informed consents at enrollment. The experimental procedures were approved by the local ethics committee at the medical faculty of the University of Tübingen and conducted in accordance with the Declaration of Helsinki. One participant took part in Experiment 1 and the other one in Experiment 2; see below for the detailed experimental procedures. For TMS neuronavigation, high-resolution 3D T1-weighted magnetic resonance images were acquired on separate sessions prior to the TMS experiments as explained in the following sections.

#### Experiment 1: TMS at Primary Motor Cortex

These data were used originally in Metsomaa et al. (2021) to study cortical excitability. A TMS stimulator (PowerMAG Research 100, MAG & More, Munich, Germany) was used to deliver biphasic pulses through a passively cooled figure-of-eight coil (PMD70-pCool, 70-mm winding diameter, MAG & More, Munich, Germany). EEG was recorded with a TMS-compatible 128-channel system (NeurOne, Bittium, Kuopio, Finland). Ag/AgCl-sintered ring electrodes were placed according to the international 10-5 system in an elastic cap (EasyCap BC-TMS-128, EasyCap, Herrsching, Germany). Impedances of all electrodes were kept below 5 k$\Omega$. Electromyography (EMG) responses were recorded simultaneously using a 24-bit biosignal amplifier in DC mode (NeurOne, Bittium, Kuopio, Finland) at a sampling rate of 5 kHz. Motor evoked potentials (MEPs) were recorded from the abductor pollicis brevis (APB) and first dorsal interosseous (FDI) muscles of the right hand in a bipolar belly-tendon montage using adhesive hydrogel electrodes (Kendall, Covidien, Dublin, Ireland). Head position was maintained using a vacuum pillow (Vacuform, Salzbergen, Germany), the TMS coil was positioned using a mechanical arm (Fisso, Baitella, Zürich, Switzerland). A stereoscopic neuronavigation system (Localite, St Augustin, Germany) was used to co-register the participant’s head to an individual MR image, to record the locations of the EEG electrodes on the scalp, and to real-time monitor coil positioning throughout the experiment.

The hand representation of left M1 was targeted orienting the coil such that the strongest field was induced in a posterior-lateral-to-anterior-medial direction. For fine tuning the coil positioning, the motor hotspot was defined as the position and orientation of the coil resulting in the largest MEP amplitudes in the right APB. Resting Motor Threshold (RMT) was determined as the minimum stimulation intensity eliciting MEPs with an amplitude of at least 50 $\upmu$V peak-to-peak in 50% of stimulation pulses (Rossini et al. 1994; Groppa et al. 2012). After preparing EEG, EMG, and neuronavigation, and recording the location of the EEG electrodes, the hotspot location was determined. The participant was seated comfortably and instructed to fixate on a visual target (fixation cross approximately 1 m in front of them). 1000 single TMS pulses were delivered with an interstimulus interval of 2 ± 0.25 s at a stimulation intensity of 110% RMT. In total, this visit lasted for about 3 h.

#### Preliminary Preprocessing of the Data from Experiment 1

We extracted the data in the epochs of [$-$ 1.5 s, 1.5 s] centered around the TMS pulse. Next, robust detrending was used to remove low-frequency drifts. Robust detrending works by cutting out all the outlier data segments (such as TEPs and artifacts) and fitting a low-frequency trendline using the remaining signal in each channel and epoch. Finally, the fitted trendline is subtracted from the original data, including the outlier segments. See de Cheveigné and Arzounian (2018) for robust detrending, and Hernandez-Pavon et al. (2022) for the description of how it can be applied to TEP data. Here, the data segments of [$-$ 4 ms, 600 ms] were preset as outlier segments to be sure that all of the TEPs were included in this interval.

**Fig. 1:**
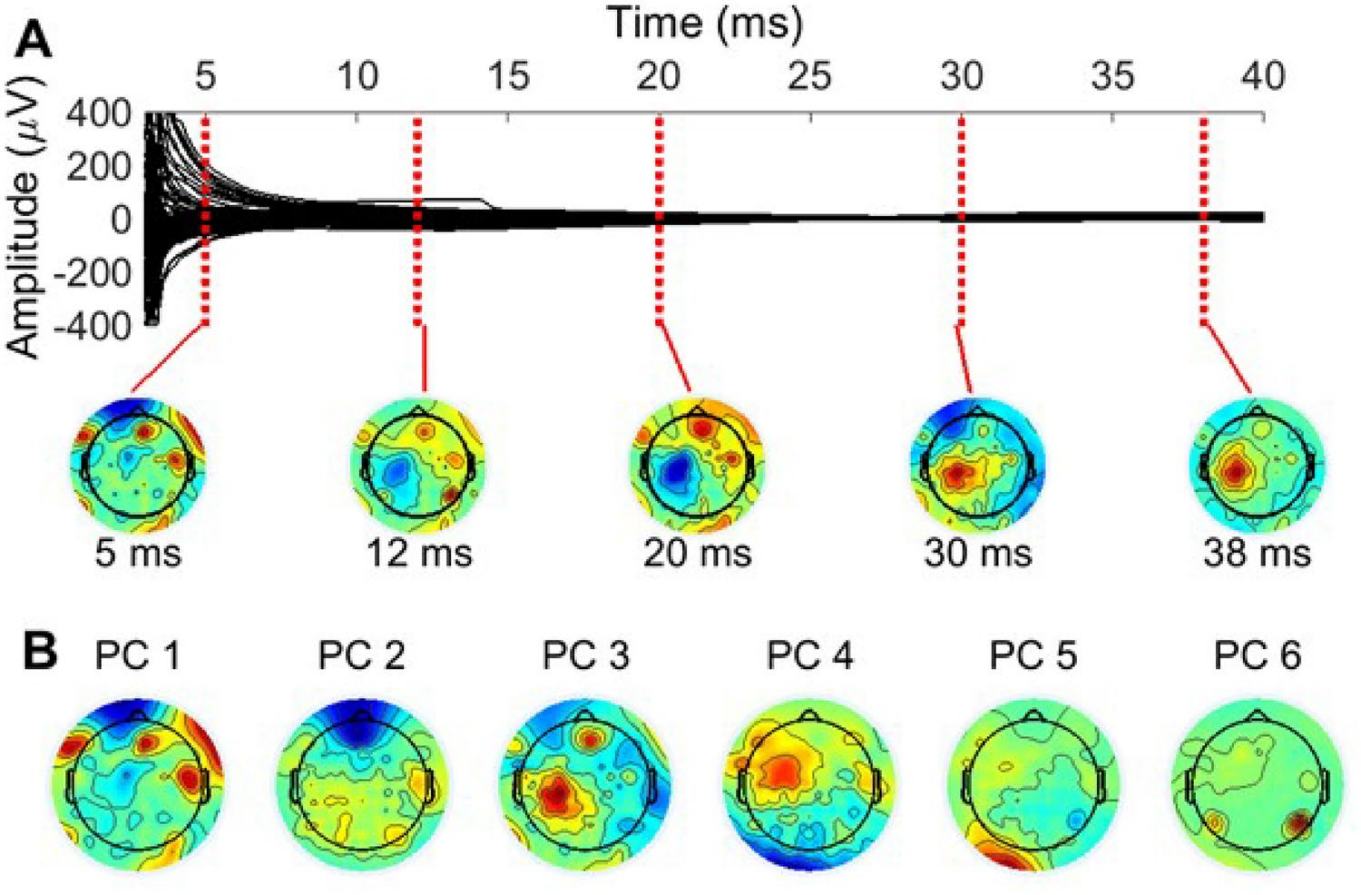
**A** Averaged TMS-evoked EEG due to primary motor cortex stimulation after detrending and averaging over all preserved trials. The 116 preserved channels are shown here from the recording described in “Experiment 1: TMS at Primary Motor Cortex” section. TMS was applied at 0 ms, after which prominent spikes and a decay artifact can be seen lasting up until about 25 ms after the pulse. **B** PCA was used to obtain topographies which contain most of the signal power of the rapidly changing EEG data. The first six topography components (PC 1, ..., PC 6) were chosen to span the estimated artifact subspace. These topographies mostly capture the non-smooth spatial maps of extracranial origin, but some resemblance to neuronal topographies can also be seen in PCs 3 and 4

As we wanted to use Laplacian trendline fitting for indentifying slow-frequency trend in the extracted EEG epochs (Metsomaa et al. 2021), **Appendix A.1**, we had to replace the outlier EEG segments, by smoothly behaving signals. For this purpose, interpolation was used. The interpolated data are not correct data, but they only serve to carry the information of smooth transition from the pre-stimulus period to post-TEP data. Laplacian trendline fitting is then able to identify the slowly changing drifts. Finally, the trendline was subtracted from the original (non-interpolated data). Since Fourier-based interpolation preserves the frequency spectrum, we resorted on this interpolation methods, as implemented in the Matlab function ’interpft’.

Thereafter, the bad channels and trials were removed. The same automatic process was used as in Metsomaa et al. (2021): First, channel-wise noise levels were estimated with DDWiener, and then, the noisiest channels were removed. The channel- and trial-wise range values of the noise estimates were used to detect the bad trials. As the statistics of the noise are changing dynamically (non-stationary noise) after TMS pulse, DDWiener was run in sub-segments of 15 ms at a time with non-overlapping windows. We considered the data within each time window across all trials stationary, so we concatenated the data for a specified time window at a time, estimated the respective noise, and slid the window forward. The early averaged TEP after the preliminary preprocessing steps, and the six first PCA components used later in removing TMS-elicited artifact by beamforming, are shown in Fig. 1. We see the pulse artifact in the very beginning of the epoch, whereafter the decay artifact lasts until about 25 ms.

#### Experiment 2: Realistic Sham TMS Versus Real TMS at Supplementary Motor Area

**Fig. 2:**
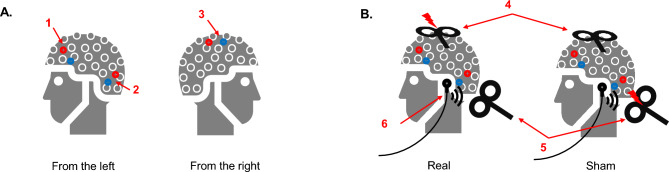
Schematic illustration of the optimized sham TMS procedure. **A** Electrode positions for electric stimulation. Three pairs of short-distance bipolar electrodes were attached to the EEG cap to deliver electric stimulation concomitantly with TMS pulses. Pairs 1 and 2 were placed in the left hemisphere, and pair 3 was in the right. The red and blue dots represent the anode and cathode, respectively. The polarity of the current was reversed after each pulse. **B** Experimental setup for the real and sham conditions. Two identical TMS coils (4 and 5) were used: One (4) was positioned on the participant’s head, aiming to target SMA with a lateral to medial induced current. The other one (5) was placed a short distance away from the head, aiming to produce the ‘click’ sound with negligible magnetic field influence on the cortex. Coils 4 and 5 were active in real and sham conditions, respectively. Concomitant electric stimulations (three pairs, intensity 24 mA, width 200 $\upmu$s) and masking noise were applied in both conditions. EEG Cap - the Noun Project icon by CIV is licensed under CC0 (https://thenounproject.com/icon/19246, https://commons.wikimedia.org/w/index.php?curid=64906229)

The measured data were from a study investigating direct cortical EEG responses to TMS at non-primary motor areas with a sham and real TMS design. Because TEPs are contaminated by peripheral sensory co-stimulation caused by TMS (Conde et al. 2019), the purpose of sham condition was to control for these multiple sensory inputs. Here, supplementary motor area (SMA) was used as a target region at its stimulation is known to elicit TEPs with only small muscle artifacts (Mutanen et al. 2013). The aim was to uncover the direct cortical EEG responses to SMA stimulation by extracting the sham-evoked response from the real TMS–EEG response. The study was approved by the ethics committee of the medical faculty at the University of Tübingen. The data were recorded from a healthy female participant (age 23) who gave informed consent at enrollment.

The TMS stimulator (Magstim 2002, Monophasic mode, UK) delivered monophasic pulses through a figure-of-eight coil (external diameter = 90 mm). 64-channel EEG signals were filtered (DC-1250Hz), recorded at a 5 kHz sampling rate, and referenced to the channel CPz online. EEG electrode impedances were kept below 5 k$\Omega$. Localite navigation system was used to plan and monitor coil positioning. After fitting the participant’s MR image into the Talairach coordinate system, the localization for SMA was defined with its MNI coordinates ($-$ 2, $-$ 7, 55). The TMS coil was placed tangentially to the gyrus, with the maximal induced current flowing approximately from lateral to medial.

To control for peripheral sensory co-stimulations caused by TMS, we applied an optimized sham procedure adapted from our previous research (Gordon et al. 2021). An illustration of the procedure is shown in Fig. 2. A sham condition was designed to simulate the somatosensory and auditory inputs as in real TMS. To reproduce the somatic sensation, we applied electric stimulation (ES) pulses to the scalp with a stimulator (Digitimer DS7A, Digitimer Ltd. UK) through short-distance bipolar electrodes. A TMS coil was placed a short distance away from the participant’s head to generate the ‘click’ sound. Additionally, masking noise was used to suppress the auditory inputs (Massimini et al. 2005). In the real TMS condition, we stimulated SMA with an intensity of 120% RMT. The TMS coil positioning was defined as described above. Masking noise was played throughout the stimulation. Importantly, ES was also applied concomitantly, aiming to saturate the somatosensory inputs so that the sensory inputs caused by TMS became negligible. Consequently, we aimed to match the peripheral evoked potentials in sham condition with those of the real TMS. To achieve this goal, the ES in this study was given at a high intensity of 24 mA (width 200 $\upmu$s) through three pairs of electrodes. With this design, we recorded 150 pulses for each real and sham condition. Finally, the direct cortical EEG responses to SMA stimulation by TMS can be revealed by removing the sham-evoked response from the real TMS–EEG response.

#### Preliminary Preprocessing of the Data from Experiment 2

**Fig. 3:**
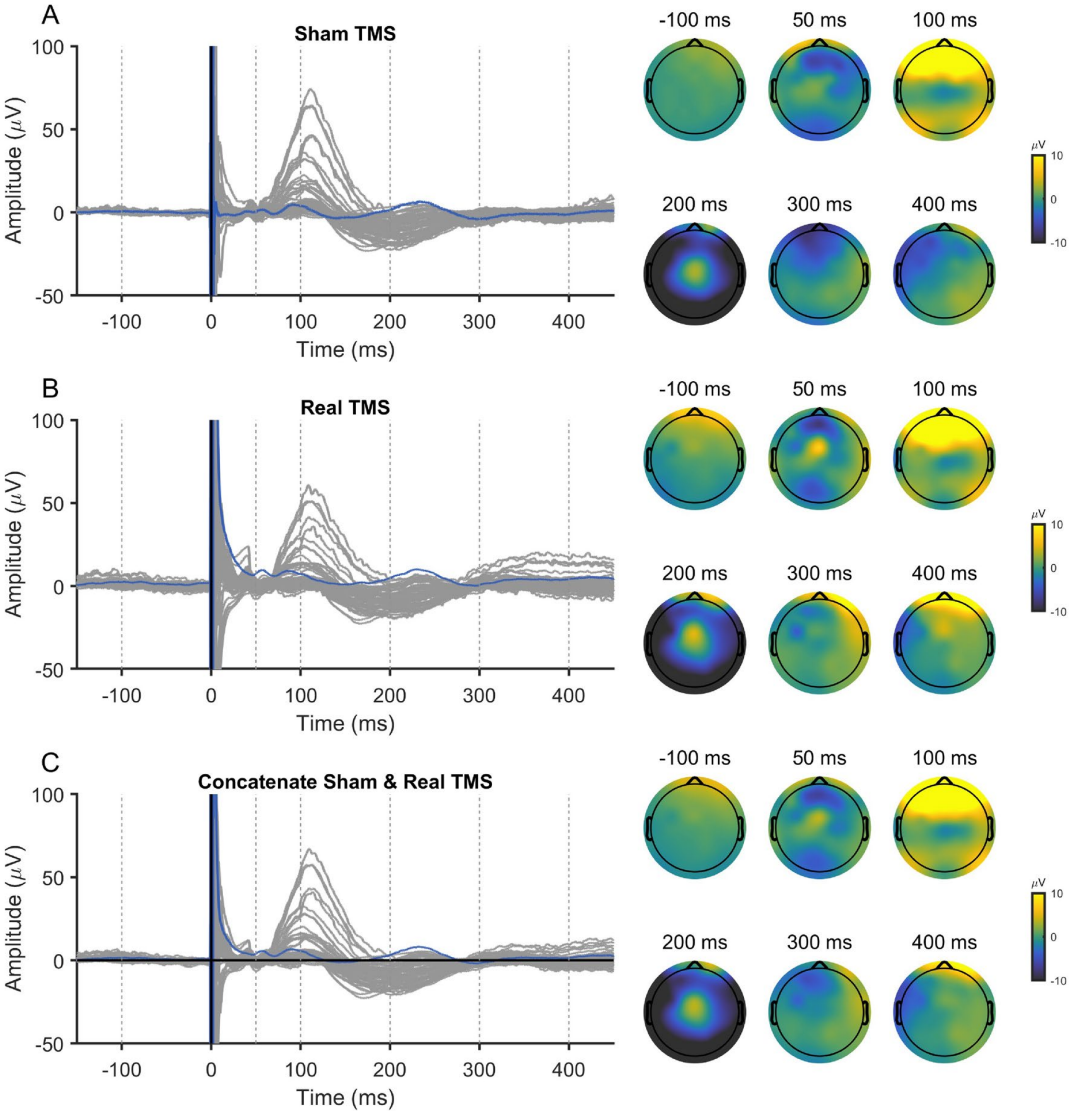
Evoked EEG responses recorded in Experiment 2: **A** the sham TMS condition, **B** real TMS condition, and **C** the grouped data, where both conditions were joined together

**Fig. 4:**
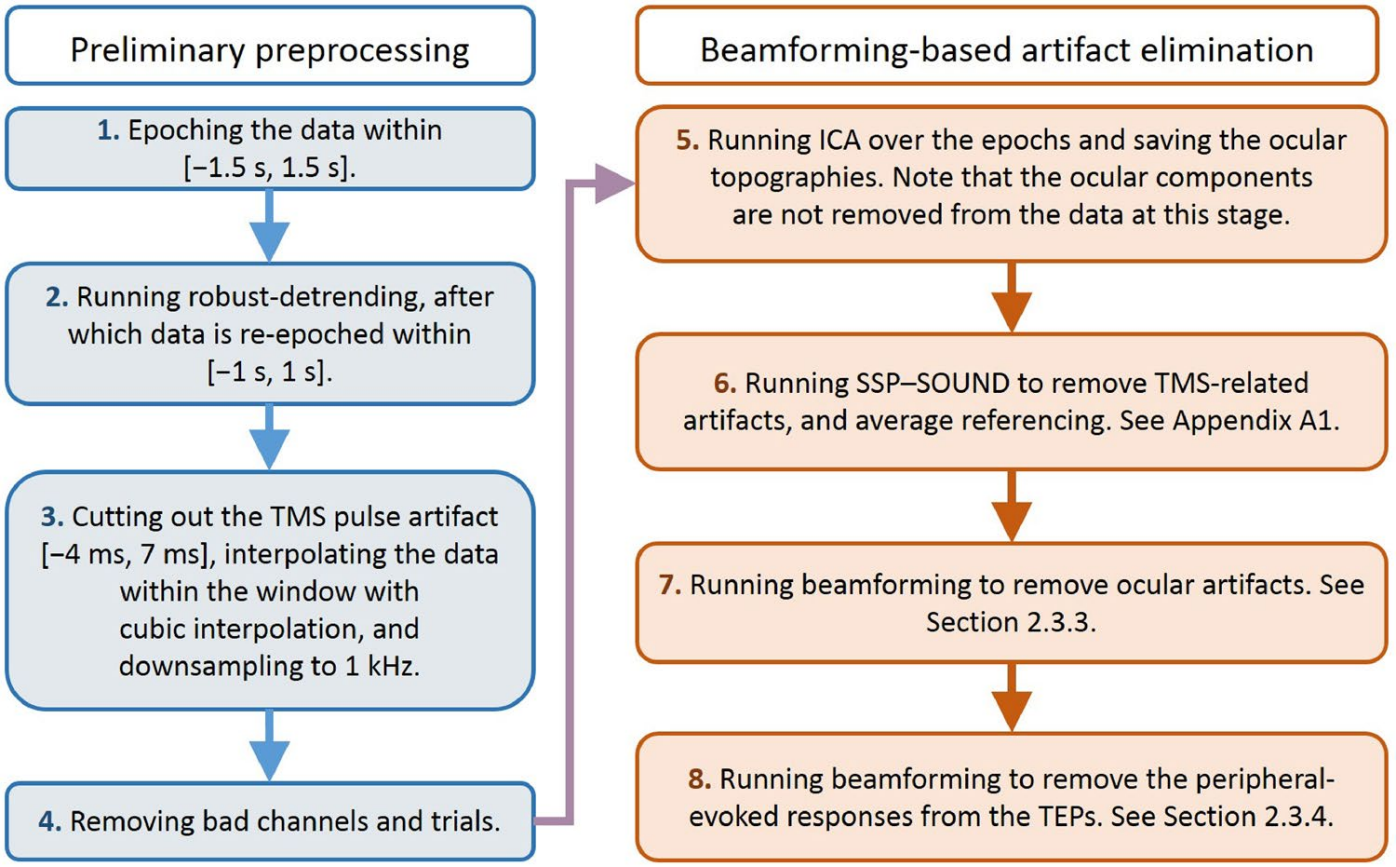
Data preprocessing and cleaning flowchart for data from Experiment 2. On the left-hand side, the preliminary preprocessing steps are listed, which are explained in “Preliminary Preprocessing of the Data from Experiment 2” section. On the right, the spatial-filtering steps are designed to eliminate three types of artifacts: (1) ocular, (2) TMS-evoked non-neural artifacts, and (3) peripheral-evoked responses. They are explained in detail in the Sections listed after each step

The entire preprocessing/cleaning pipeline applied to these data are summarized in Fig. 4. To ensure consistent cleaning for real and sham conditions, the two data sets were jointly cleaned using exactly the same operations. Therefore, we grouped the measured 150 real and 150 sham trials together; see Fig. 3 for depictions of the date from separated and joined conditions. Steps 1–4 are the preliminary preprocessing steps, and they proceed as follows: (1) We epoched the data around the TMS pulse in the interval of [− 1500 ms, 1500 ms] and baseline-corrected them using the time window of − 1000 ms to − 5 ms. (2) Slow drifts were removed through robust detrending (order = 3 polynomial fitting)(de Cheveigné and Arzounian 2018), and edge artifacts were cut off by re-segmenting the data into shorter epochs of [− 1000 ms, 1000 ms]. (3) TMS and ES pulse artifacts, from − 4 to 7 ms, were removed and cubic-interpolated before downsampling to 1 kHz (Matlab ’resample’ function was used which includes low-pass filtering at the Nyquist frequency). (4) Data were then visually inspected; channels and trials heavily contaminated by noise or artifacts were excluded manually.

Steps 5–8 are based on spatial filtering by beamforming, and they are described in more detail in the sections listed after each respective step in Fig. 4 (2.3.3., 2.3.4., and Appendix A1). In step 5, FastICA was applied to manually identify the ocular artifact topographies by visual inspection; see Hyvärinen (1999) for the description on FastICA. Note that we simply saved the topographies at this stage, and the removal was applied later. In step 6, the SSP–SOUND joint algorithm was then used to estimate the signal subspace containing the TMS-related artifacts and to suppress them from EEG signals (see “Appendix” for detailed description). In step 7, the data were re-referenced to the average, and ocular artifacts were corrected with the beamforming filter as explained in “Adaptive Cleaning with Non-stationary Data Covariance or Changing Artifact Patterns” and “Case 3: Removing Ocular Artifacts After Modifying the Data by Intermediate Processing” sections. In the final step, number 8, the real and sham stimulation condition data sets were again separated to remove the peripheral-evoked activity (represented by the sham condition) from the TMS-evoked potentials.

### Analysis of the Measurement Data

All analysis and visualizations of the EEG datasets were performed with customized MATLAB scripts (MATLAB 2019b, The MathWorks) and the EEGLAB toolbox (2020a, (Delorme and Makeig 2004)). In the following subsections, we give four different types of spatial filtering approaches based on the beamforming framework to clean evoked EEG from (1) TMS-related artifacts, (2) channel-wise noise, (3) ocular signals, and (4) peripheral-evoked potentials. The first two cleaning cases were demonstrated with data recorded in Experiment 1, and the latter two cleaning cases with data recorded in Experiment 2.

#### Case 1: Eliminating TMS-Related Artifacts

The rapidly changing TMS artifacts have very high gradient values (derivatives) as a function of time, whereas brain EEG signals are changing smoothly. Therefore, as explained in “Estimating Artifact Topographies” section, we highlighted the spiky artifacts of the data from Experiment 1 by a simple gradient estimation step. We constructed the temporal difference filter matrix according to Eq. (9) and multiplied the data by it from the right. This idea was an adapted version from Mäki and Ilmoniemi (2011), where high-pass filtering was used. We want to emphasize that any type of temporal filter can be used here provided that it serves to efficiently enhance the artifact amplitude with respect to the neuronal EEG. PCA was then used to obtain topographies with the highest eigenvalues (signal power).

With this set of artifact topographies $\varvec{\hat{A}}_\textrm{art}$, and the sample covariance matrix estimate $\hat{\Sigma }^{\textrm{sample}}$ from Eq. (6), we estimated the beamforming filter [Eq. (5)], and finally, the cleaning matrix by Eq. (4). This cleaning matrix was applied to the averaged TEPs.

#### Case 2: Implementation of Fast SOUND via Beamforming

SOUND has been introduced in Mutanen et al. (2018) to reduce the sensor noise, i.e., noise which is uncorrelated across all sensors. The algorithm makes use of an iterative approach, where the signal in each channel is linearly predicted using the rest of the channels. Noise is estimated as the non-predicted part (error term) of the sensor-signal, and the noise covariance matrix is iteratively updated based on the noise estimates. Due to its iterative nature, the run-time of SOUND may get slow with large data sets.

As derived in Hernandez-Pavon et al. (2022), SOUND may be seen as a beamforming-based cleaning approach. Here, we derive the implementation explicitly and test the run times and correctness of the outcome by comparing with the previous version of SOUND.

SOUND makes use of the model-based data covariance matrix, in Eq. (7), taking into account the neuronal and noise terms as

17 $$\begin{aligned} \hat{\Sigma }^\textrm{model}=\textbf{A}_\textrm{brain}\Lambda _\textrm{brain}\textbf{A}_\textrm{brain}^\mathrm T+\textbf{A}_\textrm{noise}\Lambda _\textrm{noise}\textbf{A}_\textrm{noise}^\mathrm T=\lambda _\textrm{brain}\textbf{L}_\mathrm {}\textbf{L}_\mathrm {}^\mathrm T+\textrm{diag}(\lambda _\textrm{noise,1},\ldots ,\lambda _{\textrm{noise},M}), \end{aligned}$$

where we use the lead-field matrix $\textbf{L}$ as the neural mixing matrix $\textbf{A}_\textrm{brain}$, and the neural sources are identically and independently distributed making $\Lambda _\textrm{brain}=\lambda _\textrm{brain} \textbf{I}$. The topographies in $\textbf{A}_\textrm{noise}$ are simply unit basis vectors $\textbf{e}_i=[0,\; \ldots ,\;0,\; 1,\;0,\;\ldots ,\;]^\textrm{T}$, one for each sensor *i*, where the *i*th element of the vector equals to 1, reflecting uncorrelated noise measured by each electrode. The noise components (sensor-wise noise signals) are uncorrelated but with different variances, making their covariance matrix diagonal $\Lambda _\textrm{noise}=\textrm{diag}(\lambda _\textrm{noise,1},\ldots ,\lambda _{\textrm{noise},M})$.

Choosing the variance of the neural sources $\lambda _\textrm{brain}$ is somewhat arbitrary, it corresponds to choosing the regularization factor in Tikhonov regularization, where the regularization factor $\lambda _\textrm{noise} / \lambda _\textrm{brain}$ is interpreted as the noise-to-signal ratio. Several heuristic decision-making rules have been suggested for choosing this parameter; see Mutanen et al. (2018) for suggestions. Here, we chose lambda, such that its inverse, referred to as the regularization factor, is $1/ \lambda _\textrm{brain}=0.01 \cdot \textrm{trace}(\Sigma )$. The same rule was used for defining the regularization factor when applying the conventional SOUND algorithm.

Note that, due to the uncorrelatedness assumption of SOUND, the beamforming filters are computed separately for each noise component, $\textbf{e}_i$, corresponding to channel *i*. On the contrary, if we allowed any correlation patterns for the noise over the channels, we would set $\textbf{A}_\textrm{art}=\textbf{A}_\textrm{noise}=\textbf{I}$ in (5). Such a choice would allow for arbitrary noise covariance, but it would result in judging the entire recorded EEG as noise. Restricting the noise correlation patterns is thus essential.

Inserting the model-based covariance matrix, and unit basis vectors one at a time as artifact topographies into Eq. (5) yields

18 $$\begin{aligned} \begin{aligned} \textbf{w}_{\textrm{SOUND},i}&={\Gamma }_i/\Gamma _{i,i}\; \textrm{where}\\ {\Gamma }&=(\hat{\Sigma }^{\textrm{model}})^{-1}, \end{aligned} \end{aligned}$$

and Γ_*i*_ denotes the *i*th column and Γ_*i,i*_ the element on the ith row and column of Γ. A very fast update rule for computing the noise filters for all channels without channel-wise iterations can therefore be written as

19 $$\begin{aligned} \textbf{W}_{\textrm{SOUND}}=[\textbf{w}_{\textrm{SOUND},1},\, \ldots ,\, \textbf{w}_{\textrm{SOUND},M}]=\Gamma \,\textrm{diag}((\Gamma _{1,1})^{-1}, \,\ldots , \,(\Gamma _{M,M})^{-1}). \end{aligned}$$

Because $\textbf{w}^\textrm{T}_{\textrm{SOUND},i}\textbf{X}$ estimates the noise in channel *i*, $\textbf{W}_{\textrm{SOUND}}=[\textbf{w}_{\textrm{SOUND},1},\, \ldots ,\, \textbf{w}_{\textrm{SOUND},M}]$ can be used to obtain the all-channel noise data by $\textbf{X}_\textrm{noise}=\textbf{W}_{\textrm{SOUND}}^\textrm{T}\textbf{X}$. For SOUND iterations, we need the noise covariance matrix, which we obtain as the sample estimate: $\Lambda _\textrm{noise}=1/T\cdot \textbf{X}_\textrm{noise}\textbf{X}_\textrm{noise}^\textrm{T}=\textbf{W}_\textrm{SOUND}(1/T\cdot \textbf{X}_\mathrm {}\textbf{X}_\mathrm {}^\textrm{T})\textbf{W}_\textrm{SOUND}^\textrm{T}=\textbf{W}_\textrm{SOUND}\textrm{Cov}(\textbf{X})\textbf{W}_\textrm{SOUND}^\textrm{T}$. Because in $\Lambda _\textrm{noise}$ only the diagonal values are non-zero, the estimate finally simplifies into

20 $$\begin{aligned} \begin{aligned} {\Lambda }_\textrm{noise}^\mathrm {}&=\textrm{diag}(\textbf{w}_{\textrm{SOUND},1}^\textrm{T}\textrm{Cov}(\textbf{X})\textbf{w}_{\textrm{SOUND},1},\ldots ,\textbf{w}_{\textrm{SOUND},M}^\textrm{T}\textrm{Cov}(\textbf{X})\textbf{w}_{\textrm{SOUND},M}) . \end{aligned} \end{aligned}$$

In the data-driven version, called DDWiener, the data covariance for filter construction by Eq. (18) is computed as a sample-based estimate $\hat{\Sigma }^\textrm{sample}=\textrm{Cov}(\textbf{X})$, yielding $\textbf{w}_{\textrm{DDWiener},i}$. Inserting $\textbf{w}_{\textrm{DDWiener},i}$’s into Eq. (20) instead of the SOUND filters, we get an alternative data-driven estimate for the noise covariance matrix:

21 $$\begin{aligned} {\Lambda }_\textrm{noise}^{\textrm{DDWiener}}=&\textrm{diag}((\Gamma _{1,1})^{-1}, \,\ldots , \,(\Gamma _{M,M})^{-1}),\,\, {\text{where}} \\ \Gamma=&[\mathrm{Cov}(X)]^{-1}.\end{aligned}$$

This noise covariance estimate is very fast to compute, and it can also be used to get an initial guess for noise covariance matrix at the start of the SOUND iterations, which are outlined in the following.

Overall, the SOUND iterations using the beamforming formulation proceed as

1. Give an initial guess for $\Lambda _\textrm{noise}$ for example by Eq. (21) where $\Gamma =(\hat\Sigma^{\mathrm{sample}})^{-1}$.
2. Estimate the model-based data covariance matrix by Eq. (17) with a chosen value for $\lambda _\textrm{brain}$, compute the inverse $\Gamma =(\hat{\Sigma }^\textrm{model})^{-1}$, and update the spatial filter matrix in Eq. (19) accordingly.
3. Update the noise covariance matrix by Eq. (20).
4. If the noise covariance has not converged, proceed to 2. Otherwise, stop iterations.

Using Eqs. (19) and (20), and (21) requires inverting $\hat{\Sigma }^\textrm{model}$ only once to update the noise levels across all channels, whereas in the original version, a new matrix inverse for each channel update was required.

As explained in detail in Mutanen et al. (2018), the final SOUND cleaning is performed using source-estimation as an intermediate step to estimate EEG due to brain activity as:

22 $$\begin{aligned} \hat{\textbf{X}}_\textrm{brain}=\lambda _\textrm{brain}\textbf{LL}^\textrm{T}(\lambda _\textrm{brain}\textbf{LL}^\textrm{T}+\Lambda _\textrm{noise})^{-1}\textbf{X}=\lambda _\textrm{brain}\textbf{LL}^\textrm{T}(\hat{\Sigma }^\textrm{model})^{-1}\textbf{X}, \end{aligned}$$

where $\hat{\Sigma }^\textrm{model}$ is obtained by the final estimate from Eq. (17), which completes the sensor-noise cleaning by SOUND.

We ran both the BF-based and conventional SOUND using 30 iterations over 116 channels with the data from Experiment 1, and measured the run times of the iterations. We also computed the differences between the sensor noise level estimates between the two versions. Similarly, with DDWiener, both the run times and the noise level differences were recorded. Since DDWiener executes only once over the channels, we performed 100 test runs to get the averaged run time.

#### Case 3: Removing Ocular Artifacts After Modifying the Data by Intermediate Processing

We used the BF-based filtering to remove ocular EEG from TEPs. The goal is to remove ocular artifacts using the topographies obtained by ICA and saved as columns in the mixing matrix $\hat{\textbf{A}}_\textrm{ocular}$. We assumed that eye movements take place randomly such that the independence assumption, required by ICA, holds. If we now used Eq. (5), to compute the spatial filters, the outcome would simply be the ICA demixing matrix (Hernandez-Pavon et al. 2022). However, this is not the goal here. Instead, an intermediate denoising step is performed, as explained below.

After performing steps 1–4 in the preprocessing Fig. 4, we ran ICA to get the topographies representing vertical and horizontal eye movements, which were saved for later use. Note that ocular artifacts were not removed at this stage, instead after estimating the topographies, we applied the SSP–SOUND cleaning to the data (see Appendix A.1) to remove TMS-induced artifacts from the evoked EEG.

ICA yields the topographies more correctly before SSP–SOUND because the latter method smooths the topographies of the underlying components, making the mixing matrix more ill-conditioned, and thus, ICA-derived topographies become prone to errors (Hernandez-Pavon et al. 2012). On the other hand, the reason for not correcting for the ocular artifacts before SSP–SOUND was that, before SOUND, any spatial filtering has the effect of mixing the sensor noise across channel. The spatial mixing makes this noise correlated over sensors, after which the noise covariance is no longer diagonal.

SSP–SOUND returns cleaning matrix $\textbf{M}_\mathrm {SSP-SOUND}$, which was used to denoise the data $\textbf{X}$ as $\mathbf {M_\mathrm {SSP-SOUND}X}$. To proceed with removing the ocular artifacts from these denoised data based on the topographies in $\hat{\textbf{A}}_\textrm{ocular}$, both the artifact topographies and the data covariance matrix were updated according to SSP–SOUND as $\hat{\textbf{A}}_\textrm{ocular}\leftarrow \textbf{M}_\mathrm {SSP-SOUND}\hat{\textbf{A}}_\textrm{ocular}$, and $\hat\Sigma \leftarrow \textbf{M}_\mathrm {SSP-SOUND} \hat\Sigma \textbf{M}_\mathrm {SSP-SOUND}^\textrm{T}$, respectively, as reasoned in “Adaptive Cleaning with Non-stationary Data Covariance or Changing Artifact Patterns” section. Finally, we computed the spatial cleaning operator using the beamforming idea from “Beamforming-Based Data Cleaning” section by inserting the modified variables into Eqs. (4) and (5).

#### Case 4: Adaptive BF-Based Cleaning to Eliminate Peripheral-Evoked Potentials

Here, the goal was to remove the peripheral-evoked activity from TEPs collected in Experiment 2. As the sham stimulation-induced responses are assumed to contain all of the undesired peripheral-induced EEG, the artifact topographies $\hat{\textbf{A}}_\textrm{art}$ can be estimated by directly applying PCA to the sham-evoked EEG as discussed in “Estimating Artifact Topographies” section. Note, that all preprocessing steps applied to the TMS-evoked data also need to be applied to the sham condition data to preserve the topographies representing peripheral responses in both conditions equal by Eq. (1).

Because the peripheral-evoked signals change as a function of time, following the reasoning of “Adaptive Cleaning with Non-stationary Data Covariance or Changing Artifact Patterns” section, we tested an approach where the cleaning was applied adaptively in a sliding window of 6 ms as detailed in the following:

1. The first 6-ms data window was defined as starting from 7 ms and lasting till 13 ms.
2. The averaged evoked EEG response within the defined window over the sham and real (TMS) condition trials were set as $\bar{\textbf{X}}_\textrm{sham, win}$ and $\bar{\textbf{X}}_\textrm{real, win}$, respectively. The data samples (columns of ${\textbf{X}}_\textrm{sham, win}$ and ${\textbf{X}}_\textrm{real, win}$) across all trials and both real and sham conditions within this time window were collected in a sample matrix $\mathbf {X_\textrm{all, win}}$.
3. The artifact topographies for the TMS-evoked EEG within a given time window were set as the sham-evoked data within the respective time window: $\hat{\textbf{A}}_\textrm{art}=\bar{\textbf{X}}_\textrm{sham, win}$. Together, these topographies represented of the removable peripheral-evoked data (in total 6 topographies with sampling frequency of 1000 Hz).
4. Using the data within this window, the covariance matrix was computed as a sample estimate of $\mathbf {X_\textrm{all, sub}}$.
5. Finally, the cleaning spatial filter matrix was computed by Eqs. (4) and (5), where Tikhonov regularization was applied. The resulting cleaning matrix $\textbf{M}_\textrm{clean}$ was applied to the averaged real condition response as $\textbf{M}_\textrm{clean}\bar{\textbf{X}}_\textrm{real, sub}$.
6. The data window proceeded by 2 ms at the time, and the cleaning was continued starting from step 2 until the real responses were completely cleaned. Using overlapping time windows guaranteed smooth outcome signals. Due to the overlapping windows, we saved the cleaned data in 2-ms segments extracting the middle 2-ms of each cleaned data 6-ms segment.

### Simulating and Cleaning Artifactual TEPs

#### Creating Artifacts on Top of Clean Evoked Potentials

From the measured TEPs in Experiment 1, we extracted clean data epochs within the late time interval between 100 and 300 ms, where artifacts were not present, and regarded these signals as neuronal data. These data had 1001 epochs and 116 channels. Each epoch $\textbf{X}_{\textrm{brain},r}$ from trial *r* was considered clean ground truth data, on top of which simulated artifact data $\textbf{X}_{\textrm{art},r}$ were added as

23 $$\begin{aligned} \textbf{X}_r=\textbf{X}_{\textrm{brain},r}+\textbf{X}_{\textrm{art},r}=\textbf{X}_{\textrm{brain},r}+\textbf{A}_{\textrm{art}}\textbf{S}_{\textrm{art},r} , \end{aligned}$$

where we simulated the artifact mixing matrix $\mathbf {A_\textrm{art}}$, and waveform matrix $\textbf{S}_{\textrm{art},r}$ for each trial *r*. As artifact topographies, we used ICA-driven topographies estimated from the early artifactual TEPs, and normalized to unit L2-norm. To this end, FastICA was applied to the data in the interval of [7 ms, 40 ms]; see Fig. 1 for the EEG within this interval. We can see a pulse artifact within the very early time window (before 5 ms) as determined by visual inspection, and if ICA is applied to intervals including time instants from before 7 ms, the independent components are almost solely artifactual. The remaining artifacts after 7 ms, including the muscular ones, last at least till about 25 ms. The time period was extended till 40 ms to be sure that the artifacts were completely included, and to increase the sample size. We chose five topographies which resembled non-neuronal components.

**Fig. 5:**
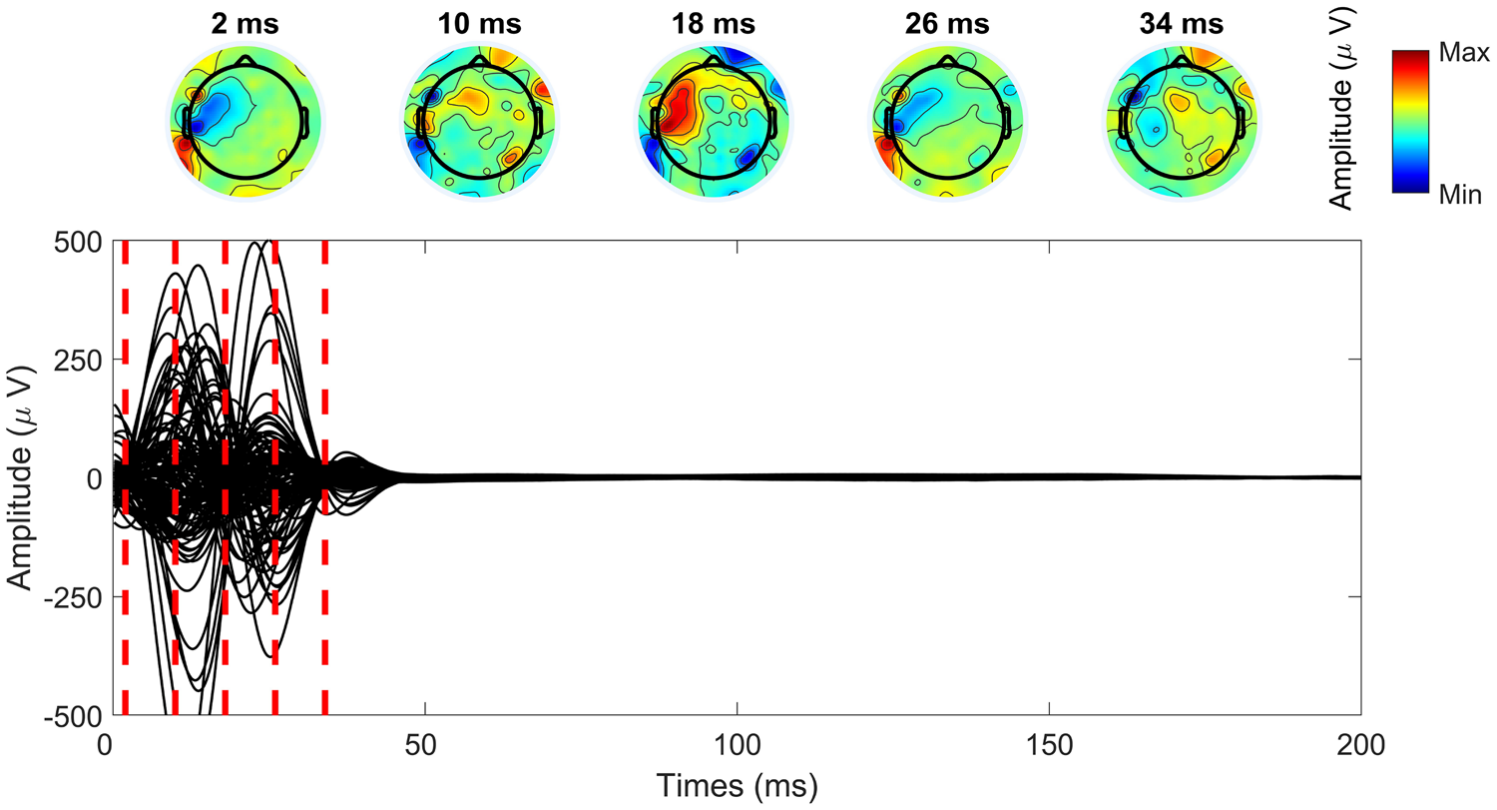
Combined data with measured clean TEPs mixed with high-amplitude artifactual simulated data within the first 50 ms. The topographies in the top row are extracted from the averaged TEPs at early latencies indicated by the red dashed lines in the butterfly plot. The clean data without artifacts can be viewed in Fig. 10

The time-courses of the five components $i=1,\ldots ,5$ were simulated as high-amplitude sinusoidal components with Gaussian envelopes:

24 $$\begin{aligned} \begin{aligned} {S_{\textrm{art},r,i}}(t)&=E(t)[0.8\cdot \textrm{cos}(\omega (t-t_{0,i}))+0.2\cdot \textrm{cos}(\omega \cdot t+\phi _r)]\, \\ E(t)&=e\cdot \exp ((t-t_{0,i})^2/\sigma ^2), \end{aligned} \end{aligned}$$

where *t* indices the time, $\omega =2\pi /70\,\text {ms}$ defines the slope of the deflections, and $\phi _r$ was random offset phase distributed evenly within $[0, \,2\pi ]$. The purpose of adding modest amount of random-phase effect was to mimic the trial-to-trial variability of the artifact. The peak-amplitude latencies $t_{0,i}$ were set from 70 ms to 490 ms with the intervals of 70 ms. The artifact life-time was defined by $\sigma =70\,\text { ms}$, and the amplitude $e=200$ $\upmu$V. In Fig. 5, an example case of the simulated data averaged over the trials is presented resulting from Eq. (23), where the high-amplitude artifacts obscure the artifact-free data. The original artifact-free data along with the cleaned outcome example are illustrated in Fig. 10.

#### Removing Simulated Artifacts and Measuring the Cleaning Accuracy

After the artifactual data were generated, we proceeded to test how well the data could be cleaned such that the resulting data would be as close to the ground truth data in Eq. (23) as possible. For cleaning, we used Eqs. (5) and (4) with both the sample-based covariance matrix ($\hat{\Sigma }^\textrm{sample}$) from Eq. (6)) and the model-based covariance matrix ($\hat{\Sigma }^\textrm{model}$) from Eq. (7) together with SVD regularization types 1 and 2, Eqs. (14), and (15), respectively. We also used a combined covariance matrix, where we summed up the model- and sample-based covariance matrices, keeping their powers (traces) equal as

25 $$\begin{aligned} \hat{\Sigma }^\textrm{combined}=\hat{\Sigma }^\textrm{sample}/\textrm{trace}(\hat{\Sigma }^\textrm{sample})+\hat{\Sigma }^\textrm{model}/\textrm{trace}(\hat{\Sigma }^\textrm{model}). \end{aligned}$$

For the BF-based cleaning, the estimated $M_\mathrm {}\times \hat{N}_\textrm{art}$ artifact mixing matrix ${\hat{\mathbf A}_\textrm{art}}$, defining the artifact subspace, was also needed. As in reality, this artifact subspace is not exactly known, we tested the robustness of the artifact suppression with variable amounts of error in the estimated artifact subspace. Erroneous estimation of ${\hat{\mathbf A}}_\textrm{art}$ can cause problems due to two reasons: (1) If the artifact subspace is underestimated, the true artifact EEG are partly not covered by the estimated subspace, and so they persist after cleaning. (2) If the artifact subspace is overestimated, all the artifacts are included, but additionally, excessive amount of clean neural EEG lies within the subspace. In the latter case, some neural signals are suppressed by the cleaning, which can happen especially when $\hat{N}_\textrm{art}$ is set greater than the true subspace dimension. Both types of errors can also take place simultaneously.

To simulate these two types of problems, we made use of two matrices to define an estimated artifact subspace ${\hat{\mathbf A}_\textrm{art}}$ with some degree of mismatch with the true subspace $\mathbf {A_\textrm{art}}$. The dimensionality of the data was fixed, as was the number of artifact components, i.e. artifact subspace dimensionality. Firstly, five orthonormal column vectors were concatenated in an $M_\mathrm {}\times 5$ matrix $\mathbf {\textbf{Q}_\textrm{art}}$, which accurately defines the true artifact subspace and would serve as the optimal choice for ${\hat{\mathbf A}_\textrm{art}}$. Note that $\mathbf {\textbf{Q}_\textrm{art}}$ has the correct dimensionality of $\hat{N}_\textrm{art}= 5$. We then allowed the dimensionality of $\mathbf {\textbf{Q}_\textrm{art}}$ to increase to $M_\mathrm {}\times \hat{N}_\textrm{art}$, where $\hat{N}_\textrm{art}> 5$, by concatenating further orthonormal column vectors. Importantly, when $\hat{N}_\textrm{art}$ exceeds five, also artifact-free part of the EEG will get included in the span of $\mathbf {\textbf{Q}_\textrm{art}}$ because our true artifact data consist of only five components. Such a choice for ${\hat{\mathbf A}_\textrm{art}}$ corresponds to overestimating the artifact subspace.

Another subspace, orthogonal to the true artifacts, was then defined. This subspace contained only neural data, and it is spanned by the orthonormal columns of an $M_\mathrm {}\times 35$ matrix ${\textbf{Q}_\textrm{brain}}$, defining the brain subspace, where the number 35 chosen based on PCA-based dimensionality reduction of the EEG. If ${\textbf{Q}_\textrm{brain}}$ was used as an estimate for the artifact subspace, none of the artifacts would get removed because they are lying entirely outside of this subspace.

In total, we distorted the estimated artifact subspace by mixing $\mathbf {\textbf{Q}_\textrm{art}}$ with variable amounts of the brain subspace as controlled by the error proportion *p*. Controlling the error in $\hat{\textbf{A}}_\textrm{art}$ was implemented by varying $\hat{N}_\textrm{art}$ and *p* in the final estimated artifact mixing matrix as

26 $$\begin{aligned} \hat{\textbf{A}}_\textrm{art}=\textbf{Q}_\textrm{art}\mathbf {\epsilon }_\textrm{art}+p\cdot \textbf{Q}_\textrm{brain}\mathbf {\epsilon }_\textrm{brain}, \end{aligned}$$

where $\hat{N}_\textrm{art}\times \hat{N}_\textrm{art}$ $\mathbf {\epsilon }_\textrm{art}$ and $35 \times \hat{N}_\textrm{art}$ $\mathbf {\epsilon }_\textrm{brain}$ contain random coefficients. Their purpose was to remix the topographies to generate different test cases for several simulation runs and to match the dimensions for summation. The random coefficients were drawn from the standard Gaussian distribution. The norms of all random coefficient column vectors were scaled to unit value.

To summarize Eq. (26), if $p=0$ and $\hat{N}_\textrm{art}=5$, the estimated artifact subspace is exactly true. As $\hat{N}_\textrm{art}$ is increased, and keeping $p=0$, the artifact subspace covers all of the true artifact dimensions, but also includes an increasing amount of the neuronal EEG subspace, which may lead to overcleaning of interesting neural data since the true artifact subspace is at a constant value of five. Moreover, when *p* is increased, the brain subspace is mixed with the true artifact subspace, leading to bias in the estimate $\hat{\textbf{A}}_\textrm{art}$. As a result, EEG gets undercorrected due to artifact leakage.

To systematically test the success of cleaning with respect to the accuracy of the estimated artifact subspace, we varied the dimension and subspace error such that $p=\{0\%, 1\%, 2\%, 5\%, 10\%, 20\%, 50\%\}$, and $\hat{N}_\textrm{art}=\{5, 6, 7, 10, 13, 17, 25\}$ in Eq. (26), respectively. We randomized the simulated data with Eqs. (23) and (24) 100 times for each combination of parameters *p*, $\hat{N}_\textrm{art}$.

After each run, the artifactual data were cleaned by Eqs. (5) and (4), with the chosen estimates for the covariance matrix and artifact subspace. The success of cleaning was measured as Relative Error (RE), i.e., power of the difference between the estimated data ${\hat{\mathbf X}}_\textrm{brain}$ and the true clean data $\textbf{X}_\textrm{brain}$ relative to the power of the true clean data:

27 $$\begin{aligned} \textrm{RE}=\Vert {\hat{\mathbf X}}_\textrm{brain}-\mathbf {X_\textrm{brain}}\Vert _\mathrm F/\Vert \mathbf {X_\textrm{brain}}\Vert _\mathrm F, \end{aligned}$$

where $\Vert \cdot \Vert _\mathrm F$ stands for the Frobenius norm that was used to compute the data power. When the estimated clean data exactly corresponds to the original one, the relative error becomes 0. RE increases as the difference grows bigger. RE was averaged across the 100 repetitions at each fixed parameter combination to get the final measure of the cleaning accuracy.

## Results

### Cleaning Various Artifact Types from Measured TEPs

#### Case 1: Cleaning TMS Artifacts

The recorded artifactual data are depicted in Fig. 1, where we see that the artifact signals are rapidly changing and approaching zero after a high peak arising right after the TMS pulse. These artifacts were suppressed as explained in “Case 1: Eliminating TMS-Related Artifacts” section. PCA of the difference-filtered data was used to yield six topographies with the highest eigenvalues (Fig. 1B), collected in $\hat{\textbf{A}}_\textrm{art}$, representing (or spanning) the artifact subspace, meaning that all artifact topographies within the artifact subspace can be presented as weighted sums of these chosen PCA topographies.

**Fig. 6:**
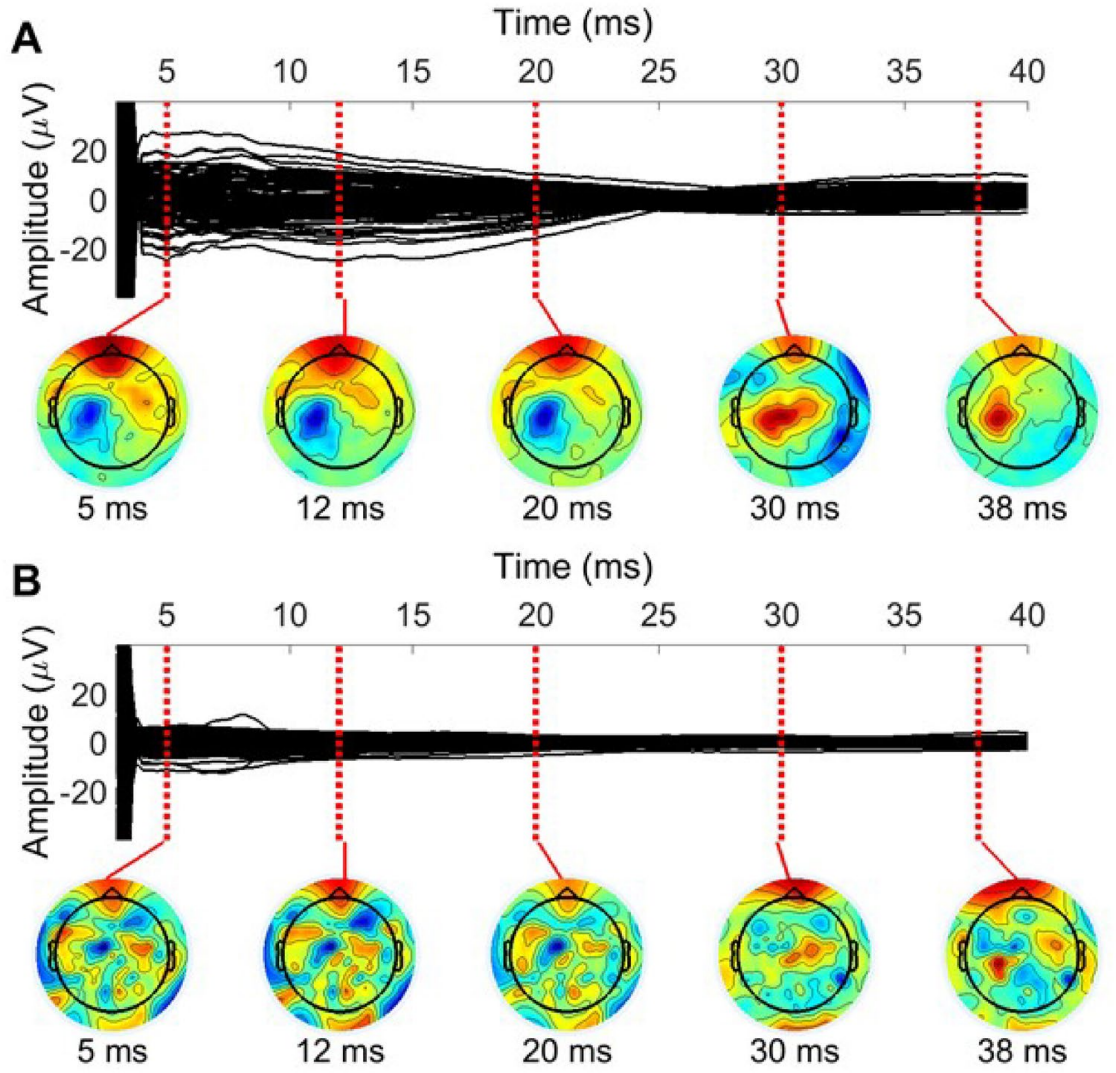
Averaged early TEP due to motor cortex TMS cleaned by two beamforming-based spatial filters. The subspace spanned by the topographies in Fig. 1 was set as the artifact space, within which the removed EEG lies. **A** Beamforming is used here with a small regularization factor (0.01). In this case, artifactual EEG data are removed such that they are lying within the defined subspace and are uncorrelated with the neuronal EEG (outside of the artifact subspace). **B** Beamforming is used here with a large regularization parameter ($10^3$), corresponding to the SSP method. In this case, more data get eliminated since all the data within the artifact subspace is deleted, regardless of correlations with the neural data

Once a set of artifactual topographies was estimated, beamforming-based cleaning was used by Eqs. (5) and (4). The sample-based covariance matrix was computed with the mean-subtraction given by Eq. (6). We used two distinctly different regularization coefficients, leading to the corrected data shown in Fig. 6. The small regularization in Fig. 6A preserved more of the data. Namely, most data in the artifact subspace, whose waveforms could be predicted by the data in the orthogonal neuronal subspace, were returned back to the cleaned data by beamforming. Note that the cleaned data still show remaining spatial patterns resembling the artifact topographies in Fig. 1B, indicating that not all of the data lying within the artifact subspace were deleted. On the contrary, large regularization in Fig. 6B, corresponding to complete out projection, is discarding all the data in the estimated artifact subspace, leading to highly attenuated signals. In addition to the small overall amplitudes after cleaning the data with heavy regularization, the remaining topographies are heavily distorted, making the visual interpretation difficult, as can be seen by comparing Figs. 1A and 6B. We note that, for EEG source modelling, one may take this distortion into account by applying the same cleaning matrix to the data and to the mixing matrix/lead-field matrix in Eq. (1). This approach is taken by the SSP–SIR algorithm, where minimum-norm estimation is applied, but can be utilized in conjunction with any source localization method.

#### Case 2: Performance of the Beamforming-Based SOUND Implementation

We compared the speed and the similarity of results when estimating the noise levels (noise covariance matrix) with the novel implementation by beamforming in Eq. (18) and the version in Mutanen et al. (2022), which is an updated version from the original one published in Mutanen et al. (2018). The comparison was made both for DDWiener (data-driven approach) and SOUND (model-driven approach). We ran the algorithms for the EEG data recorded in Experiment 1, consisting of 116 channels. For the original version of SOUND, channel-wise iterative process is performed, so the number of channels defines the execution time. The SOUND iteration (steps 2–3 in “Case 2: Implementation of Fast SOUND via Beamforming” section) was applied over all channels 30 times, and we computed the mean run time for each SOUND iteration.

The mean run time of one iteration for the novel SOUND implementation was $3.9\times 10 ^{-4}$ s, while for the previous implementation, it was 0.14 s, making the new version around 360 times faster. Typically, SOUND is run around 10 times, which increases the absolute run time difference even further. The maximum difference over the estimated channel noise levels (standard deviations of noise components) was $2.6\times 10^{-5} \;\upmu V$, which was around $4.6\times 10^{-4}$% of the mean noise levels estimated by the conventional method, making the values practically equal.

The average run time of DDWiener was $3.6\times 10^{-4}$ s as computed with the BF-based implementation over 100 repetitions. For the original implementation, the run time was 0.043 s on average, the difference of speed thus being around 120-fold. The maximum difference of the estimated channel noise levels (standard deviations) was $0.059\; \upmu V$, which was around 1.8% of the mean noise levels estimated by the conventional method.

#### Case 3: Ocular Artifact Elimination with Adaptive Cleaning

**Fig. 7:**
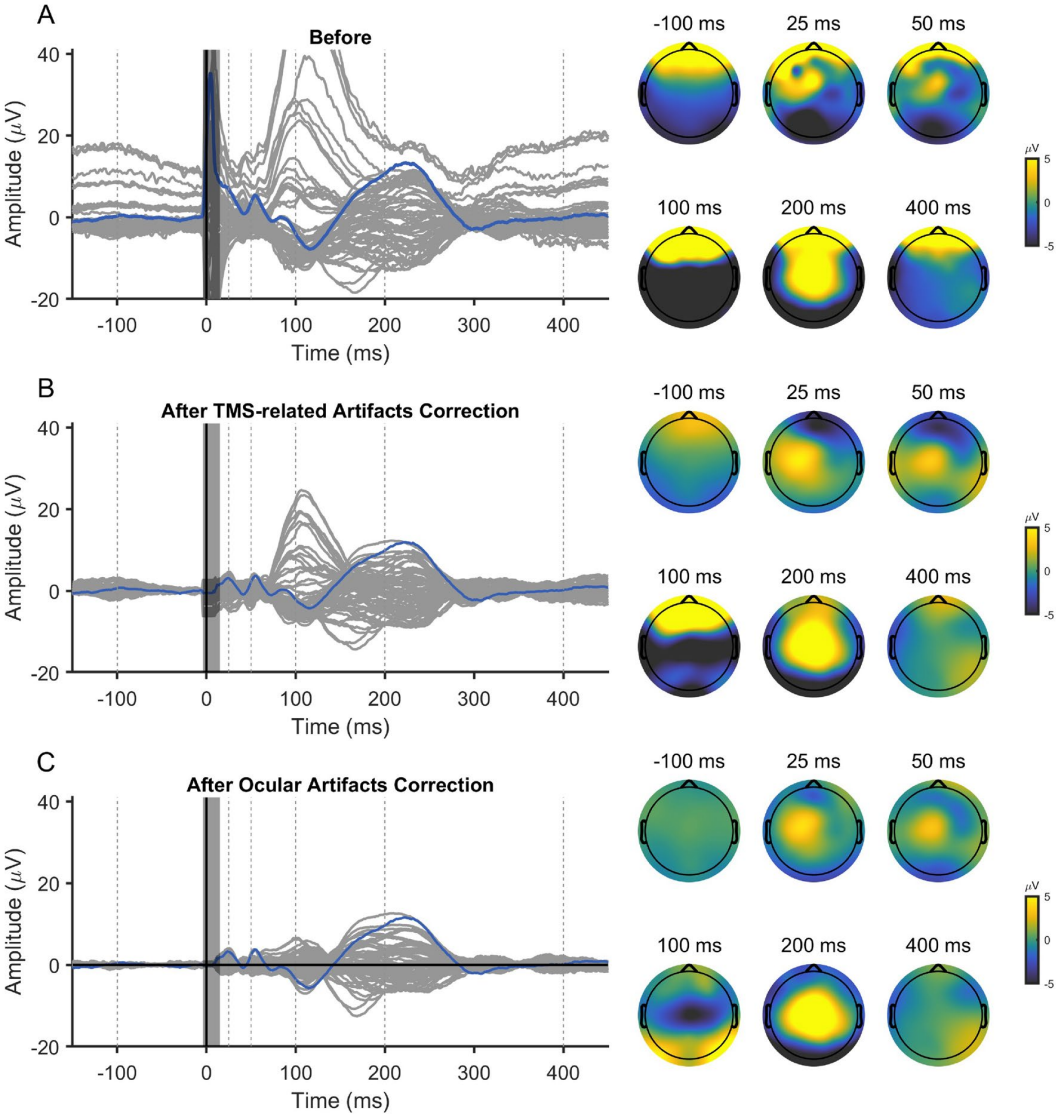
Removal of ocular artifacts by adaptive beamforming. **A** TEPs show positive blink signals predominantly in the frontal channels as seen in the topographic maps. They are present throughout the time window, but TMS-evoked blinks is seen to average at 100 ms after the pulse. **B** After TMS artifact removal, large amount of ocular EEG have been eliminated because the artifact topographies have included ocular patterns too. Due to temporal overlap, the extraction of pure TMS artifact topographies was not possible. Some ocular activity still remains as seen in the topographies and, especially, in the high-amplitude deflection at 100 ms. **C** The remaining blink artifacts are removed with the adaptive beamforming-type spatial filtering. The 100 ms and 200 ms-deflections are recovered without the blink contributions. The baseline data also stays at zero after filtering out the persisting ocular activity

Here, we demonstrate how ocular EEG artifacts may be eliminated from the data by filtering with the help of beamforming when the data are modified. ICA was used to derive the vertical and horizontal eye movement topographies, but they were not removed at this stage. SSP–SOUND (Appendix A.1) was applied to eliminate the prominent TMS artifacts. Only after this step, to suppress the ocular EEG beamforming-based cleaning filter was estimated as explained in “Case 3: Removing Ocular Artifacts After Modifying the Data by Intermediate Processing” section. The outcome is depicted in Fig. 7. In the original data (panel A), after the detrending, one can see the frontal channels being on average above the zero-level even before TMS. This reflects the fact the blinks create large-amplitude monopolar (positive) deflections at various time instants, which are averaged into somewhat positive signals in the frontal areas. The TMS pulse has a tendency to provoke blinks, which is seen here at around 100 ms, where a remarkably high-amplitude positivity is seen in the frontal area.

When removing the TMS artifacts (panel B, in Fig. 7), we see that the ocular activity also gets attenuated. This is due to the temporal overlap of the TMS artifacts and the blinks, so the estimated artifact topographies span partly the blink topographies. Still, the outcome topographies show that frontal channels demonstrate blink-related activation maps dominating especially at 100 ms, but also at 200 ms.

Finally, the ocular artifacts were removed with the adaptive beamforming -based spatial filtering as illustrated in panel C, Fig. 7, where we see that the baseline activity is steadily set to zero, and the 100 ms-deflection is cleaned from the predominantly frontal activity. This deflection is not fully nullified as we now see the non-ocular EEG remaining after extracting and eliminating the blink pattern only. Additionally, at 200 ms, we can see the topographic map showing the positivity around the vertex without misleading frontal activity.

We can also see that the early deflections at around 25 ms and 50 ms are preserved throughout the cleaning steps of removing both TMS-related and ocular artifacts. Additionally, at 100 ms, bilateral positivity emerges in temporo-occipital regions of the topography. Simultaneous central negativity suggests possible tangential bilateral sources in parietal/temporal areas, which could reflect sensory-evoked potentials evoked by the TMS stimulus. Such peripheral-evoked potentials are further removed by BF as illustrated in “Case 4: Cleaning Peripheral-Evoked Potentials” section.

#### Case 4: Cleaning Peripheral-Evoked Potentials

**Fig. 8:**
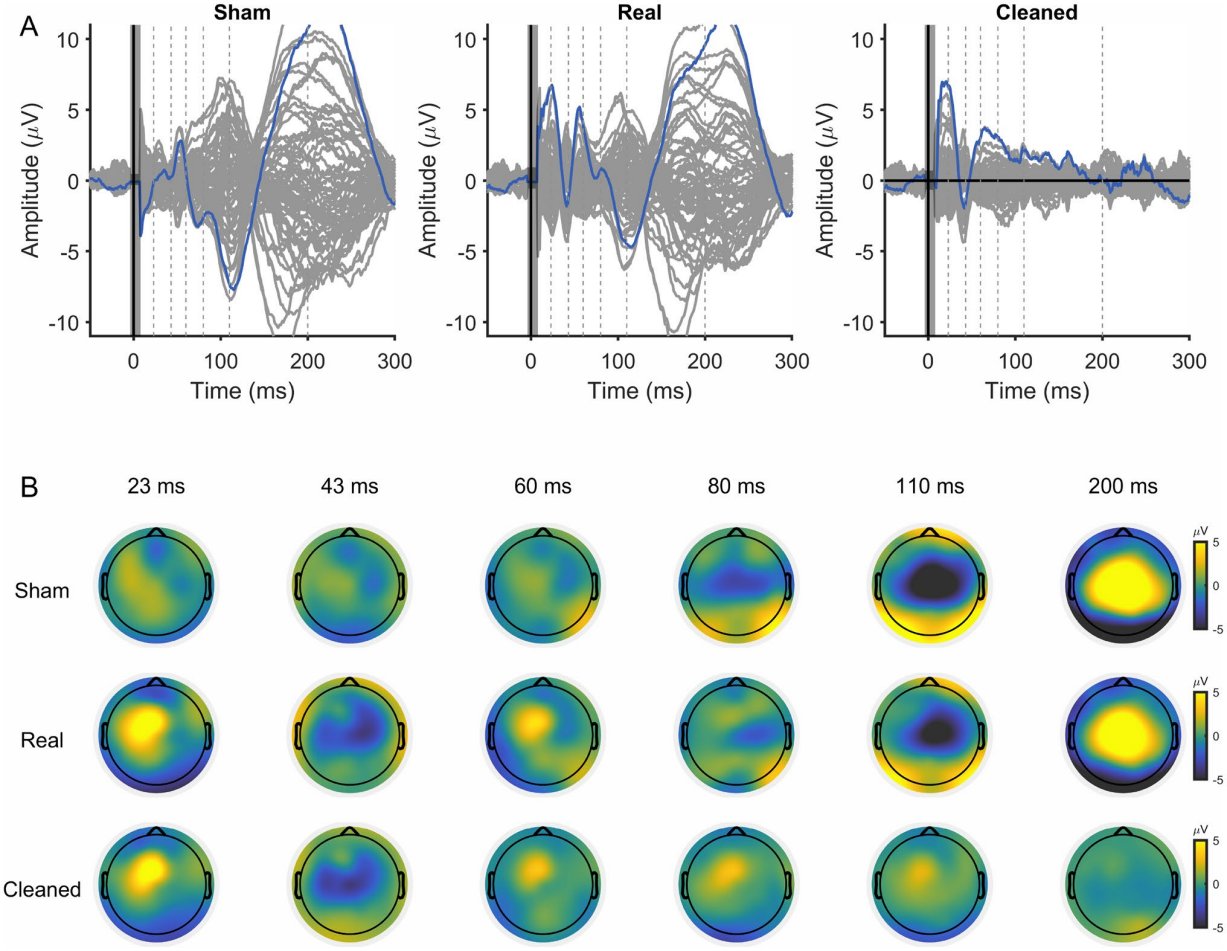
Comparison of evoked EEG responses before and after eliminating perirpheral-evoked potentials from real condition following SMA stimulation. **A** Butterfly plots show the temporal deflections; from left to right are sham, real, and corrected (cleaned) conditions after removing sham from real. Blue lines correspond to channel FCz, i.e., close to the site of real TMS. Grey shaded area corresponds to the time windows of TMS pulse artifacts. Dashed lines show the time instances corresponding to 23, 43, 60, 80, 110, and 200 ms. **B** Topographies show the spatial distribution of EEG responses at the timestamps of the dashed lines

The TMS–EEG data in sham and real were recorded as explained in “Experiment 2: Realistic Sham TMS Versus Real TMS at Supplementary Motor Area” section. The sham condition was used to capture the EEG responses resulting from peripheral sensory stimulation; see illustration of these data in Fig. 3. Assuming that the same sham response -generating neural sources were also active in the TEPs, the aim was to eliminate the components from TEPs that generated the sham topographies. Beamforming by Eqs. (5) and (4) was used to this end as outlined in “Beamforming-Based Data Cleaning” section.

The cleaned data are shown in Fig. 8. To facilitate the visualization of stimulation-evoked EEG responses, we divided the post-stimulation time windows into early (before 60 ms), middle [60 ms, 149 ms], and late [150 ms, 250 ms] intervals. We observed that the spatiotemporal patterns of EEG responses from sham condition closely resembled those in the real condition across the middle and late intervals. Two significant evoked peaks occurred at around 100 ms and 200 ms and were distributed across frontocentral and central areas. This feature corresponds to the classical N1–P2 complex (Goff et al. 1977), indicating the cortical responses evoked by auditory and somatosensory stimulations. As early as around 20 ms, positive deflections were resolved in real TMS condition and distributed across channels close to the SMA. After eliminating the sham condition evoked EEG from real with the BF-method, we can observe that the early deflections were well preserved, while the N1–P2 complex was removed to a large extent. This indicated a successful attempt to suppress multiple peripheral evoked potentials while resolving ‘true’ TMS evoked cortical responses with the BF-method.

### Simulation Results

The simulations were created as described in “Simulating and Cleaning Artifactual TEPs” section. The true simulated artifact data consisted of five components. As artifact topographies are generally not exactly known a priori, we systematically varied the dimensionality of the estimated artifact subspace (the number of PCs defining artifacts) and the error of this subspace estimation to see how robust the cleaning is with respect to these parameters.

**Fig. 9:**
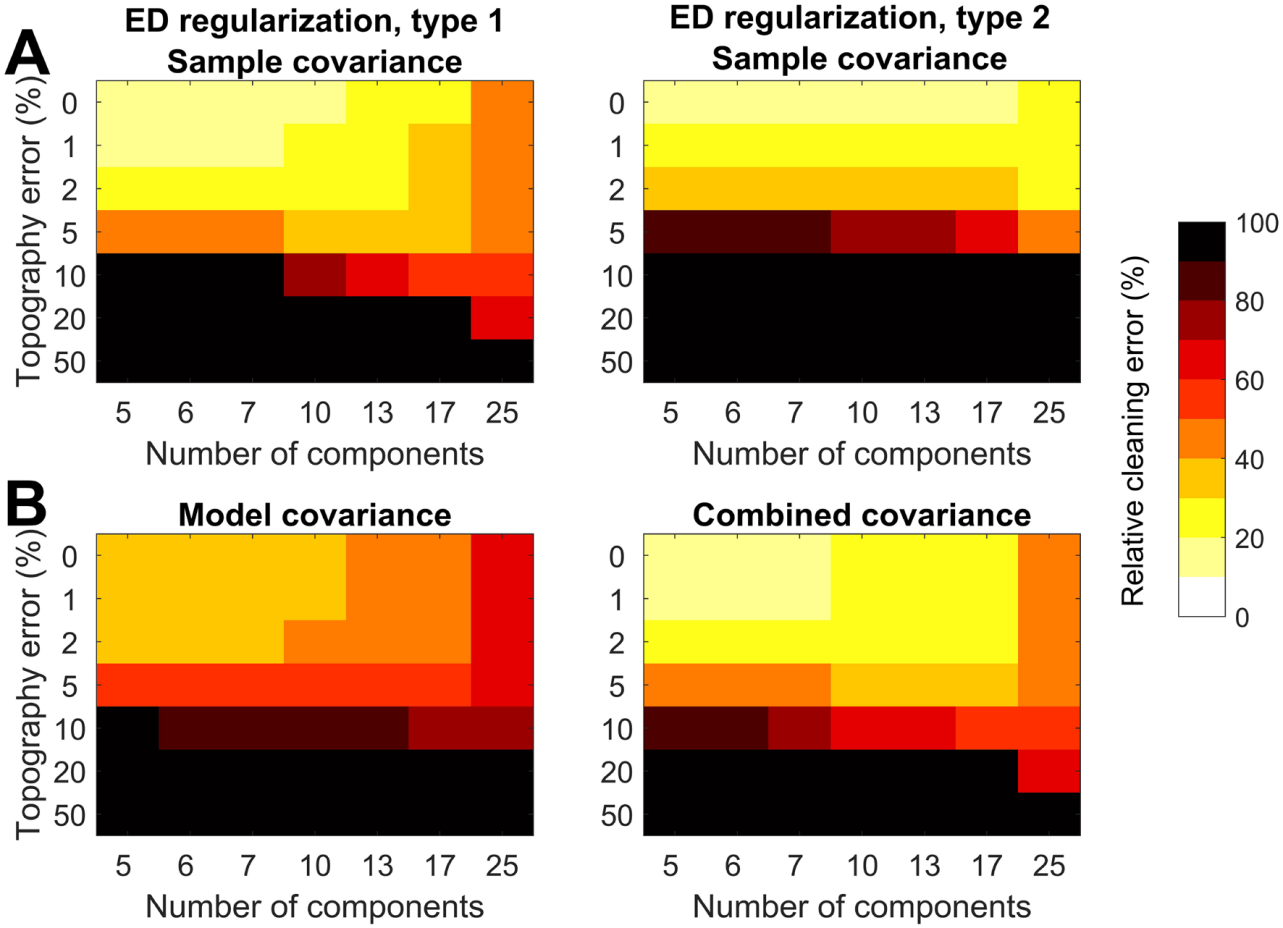
Results from cleaning simulated artifacts to estimate the original clean measured TEPs. Systematic comparison was conducted by varying the topographies defining the artifact subspace; both the number of topographies and the error of the subspace were iteratively changed to get a complete mapping of the resulting cleaning error. **A** Comparison between ED-based regularization types 1 and 2. Relative topographic error describes the bias of estimated artifact subspace compared to the true one, and the number of components indicates the dimensions of the estimated artifact subspace. Type-1 ED-truncation gives more accurate cleaning results than type-2 when the topographic error grows higher that 1%. Sample-based estimation was used to obtain the covariance matrices. **B** Model-based and combined covariance matrix estimates were also compared, as given by Eqs. (7) and (25), respectively. Model-based covariance makes use of the lead-field matrix, and the combined model is an ’average’ of the sample- and model-based estimates. The type of regularization was ED (14), with the cutoff at 35 components. The same color map is used over all panels

We tested the ED-based regularization types 1 and 2 in Eqs. (14) and (15), respectively since type 2 has not been previously used in the context of beamforming. We also performed the comparison of the BF-based cleaning using sample-based covariance, model-based covariance, and a combination of these two. For the regularization type comparison, we used the cutoff eigenvalue number of 35 when computing the pseudoinverse by ED regularization type 1. Since in type-2 ED regularization, the rank of the covariance matrix reduces by the number of artifact components $\hat{N}_\textrm{art}$, the cutoff number was reduced to $35-\hat{N}_\textrm{art}$, respectively. Sample covariance matrix was used in both cases. The comparison results are shown in panel A of Fig. 9. For small topographic errors, 0–1%, type-2 regularization gives more accurate results when the number of estimated artifact components increases. However, type-1 regularization is more robust with respect to the increasing topographic error beyond 1%.

**Fig. 10:**
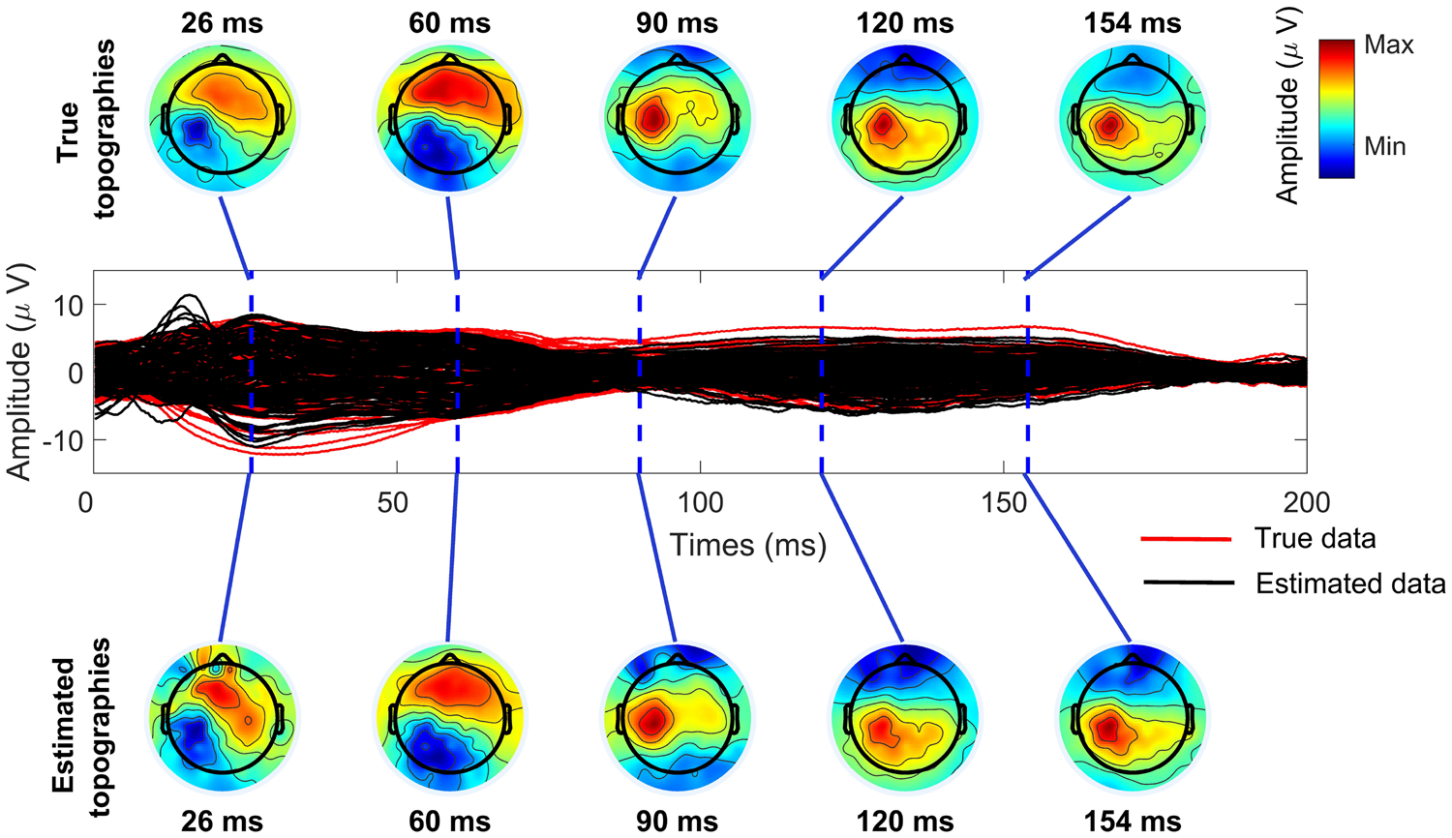
An example case of cleaning EEG where simulated artifacts were imposed on the measured clean TEPs. Here, 13 topographies were used to define the artifact subspace, while the true subspace was defined by 5 topographies. The error of the used subspace relative to the true one was 5%. The resulting relative cleaning error was 31% in this case. The topographic maps of the true clean data and those of the estimated data are depicted in the top and bottom rows, respectively. Each topography is scaled individually within the shown color map such that zero is represented by green. The topographies are extracted at the given latencies from the averaged responses of the true and estimated EEG, as shown in the butterfly plot. Note that the original data with artifacts are seen in Fig. 5

An example case of the cleaning outcome is shown in Fig. 10, where the cleaned data are overlaid on the original ground truth data (See the original artifactual data in Fig. 5.). Here, an error of 5% in the artifact subspace was used, and 13 PCs were set to span it. The resulting relative error was roughly 30%, and we can see that the estimated data have high overall correspondence with the original data. One can easily observe the locations and latencies of maximal activity. Topographies have slightly smeared due to cleaning, and the artifactual time window shows some extra small-amplitude ripples (both in time and space), which indicates a small proportion of artifact leakage. In the late time window, we can see slight attenuation in the neuronal EEG amplitudes due to modest over-correction.

Beamforming filter was then computed with three different covariance matrices: (1) sample-based covariance matrix was used according to Eq. (6), (2) the model-based was set by Eq. (7), and (3) the combination of these estimates was defined by Eq. (25). Based on the systematic comparisons of the three covariance matrix types in Fig. 9, we see that increasing the subspace dimensionality does not seem to drop the cleaning accuracy very dramatically until the number of PCs gets very high. On the other hand, the cleaning outcome is rather sensitive to errors in the artifact subspace estimation: If the error grows larger than 5%, meaning that the artifact space is no longer spanned by the PCs, the data cleaning ends up with large relative error. Due to very high amplitudes, even small fraction of leakage from the artifacts can significantly distort the data interpretation.

Both model-based and sample-based estimations behave in a similar manner as described above. The most accurate results are achievable with the sample-based and combined model-based approaches. The combined approach is slightly more robust when the subspace estimation error increases to the level of 5% and a high number of PCs is used.

## Discussion

### Benefits of the Beamforming Implementation

Different research questions and TMS–EEG data sets have different challenges and goals. It is not therefore optimal to always run the recorded EEG through the same preprocessing steps. It is poorly understood though how one should select the cleaning tools. In this work, we demonstrated that, after specifying the needs of the preprocessing and the properties of the data, one may use as a starting point one single framework for the design of the cleaning method. The methodology and results presented in this work are all based on one single formulation of beamforming. This approach greatly simplifies the implementation, comparison, and understanding the differences of the different spatial cleaning approaches as we no longer need to regard them as separate entities.

Implementing the artifact-erasing spatial filer requires choosing the best available estimate for the data covariance matrix and estimating the topographies that span the artifact subspace. From this point of view, it becomes straightforward to implement novel spatial cleaning filters adapted to the study at hand.

In addition to simplifying the spatial filter implementation, the run time of the computation reduces if multiple filters for several artifacts can be computed at once. Usage of beamforming requires inverting the covariance matrix, which is time-consuming, but this inverted covariance then stays the same even if several filters are needed for different uncorrelated (sets of) artifacts. As a result, even joint-implementation of multiple filters can end up with compact formulations. This was demonstrated with the SOUND algorithm, for which the computational time was decreased by 99.9%, when using the beamforming formulation as compared to the previous implementation.

### Guidelines for Usage

Based on our tests on removing simulated artifacts from EEG by beamforming, the best cleaning accuracy was obtained by using the sample covariance matrix estimation compared to other covariance estimation types. However, this may not always be the optimal choice as the data quality from different measurements can be quite variable. With some data sets, the sample size may also be too small to define the data covariance accurately. In these cases, defining the covariance based on the lead-field matrix can be the safest choice, i.e., Eq. (7). The results showed that this model-based estimation also provided small relative errors provided that the artifact subspace was estimated rather correctly.

The intermediate type of covariance estimation by superimposing both sample- and model-based covariance matrices can also be useful. This type has the benefit of regularizing according to the lead-field matrix possibly improving the robustness of the estimation if the sample size is not large enough, or in case of outliers in the collected data. Naturally, if some prior knowledge of the activated areas exists, it is beneficial to take this into account in the source covariance matrix to make the model-based data covariance more accurate: For example, if some areas are known not to be active the respective source variances can be set to zero.

Based on the results in Fig. 9, it can be concluded that the accuracy of the artifact topographies is the most critical part of the spatial filter estimation. We wish to emphasize though that these topographies do not need to be the true artifact topographies, but instead it is enough if the estimated topographies span the spatial subspace where the topographies lie. Moreover, this span can cover more than strictly the artifact subspace, as long as it does not fully enclose interesting neuronal EEG, so that the relevant EEG will be preserved.

If pure artifact data can be measured or extracted from the data separately, PCA is a practical tool to extract the artifact topographies. The number of PCs should be tuned such that the principal vectors cover at least the artifact subspace, so preferably too many rather than too few PCs should be included. If some segment of the cleaned data decreases close to zero, the number of PCs is probably too high.

Increasing regularization may also help in situations where it seems impossible to define the artifact subspace with a compact set of topographies: If residual artifacts remain in the data with a reasonable number of artifact topographies, one can adjust the regularization coefficient higher [Eq. (13)]. This procedure has the effect of more efficiently eliminating the artifact, with the cost of dampening the neuronal EEG simultaneously.

Comparing the results of eliminating peripheral-evoked EEG from TEPs to those of Biabani et al. (2019), it seems that beamforming-based cleaning preserves the EEG amplitude better than SSP–SIR or ICA. This can be partly simply due to the fact that, here, the cleaning was conducted within short time widows, where the artifact topographies span a smaller subspace, within which the amplitudes are suppressed. On the other hand, as described in detail in Hernandez-Pavon et al. (2022), SSP–SIR corresponds to the beamforming-based cleaning when the model-based data covariance matrix is used according to Eq. (7), where as here, we used the sample covariance matrix by Eq. (6). The first option takes into account the individual anatomy, but assumes uniform neural activity around the cortex at all times, whereas the latter option reflects the individual cortical EEG-generating processes. Thus, the amplitude differences are also likely due to taking into account individual function rather than anatomy.

### Challenges and Limitations

There are several parameters in the presented cleaning approaches which need to be manually determined. Our selections are mostly selected by visual inspection and practical observations. For example, if we included in PCA EEG samples from the time interval of [− 4 ms, 7 ms], we got almost solely artifactual components, which makes the data cleaning practically impossible. The modern amplifiers allow the EEG signal to return to baseline levels within 5–10 ms after the TMS pulse under optimized recording conditions (Rogasch et al. 2017), which matches with our observations. Similarly, with the 6 ms sliding window, we do not wish to state that our selection is the optimal one. One could also use a longer time window if one is convinced that the stationarity still applies. Lengthening the time window would be beneficial since a larger sample size improves the covariance matrix estimation, but this is achieved at the cost of including increasingly non-stationary data intervals in the estimation, which may bias the cleaning outcome. The early TEP latencies tend to have rapid changes from the negative/positive to positive/negative peaks within around 15 ms so we decided to be careful with the stationarity assumption over longer intervals (Komssi et al. 2004). Further studies would be needed to determine optimal optimal time window widths.

A major practical question for applying Eq. (4), for the cleaning operator estimation is how to estimate the artifact-subspace-spanning topographies. The simulation results suggest that accurate retrieval of the subspace is essential. With measured EEG, we illustrated different strategies for estimating the artifact topographies: (1) For ocular artifacts, we here used ICA. (2) To obtain rapidly changing artifacts, temporal difference filter followed by PCA was chosen. (3) To get peripheral-evoked data, sham stimulation protocol was used. (4) To eliminate sensor-noise noise, the artifact topographies were predetermined by definition.

Qualitatively the results are reasonable, but still it remains as an open question how to optimally estimate the subspace. Naturally, the best option would be to know the subspace a priori, but this is most often not feasible. The most popular tools for artifact topography estimation are PCA and ICA. One should be cautious with ICA because the estimated topographies easily span over neural interesting EEG when the data are time-locked to the stimulation pulse (Hernandez-Pavon et al. 2022).

We would like to point out that slowly changing signals (low-frequency data) are a major problem for spatial filtering by beamforming. This is due to the fact that, the slow processes easily create artificial correlation patterns, which leads to signal leakage between EEG components by beamforming filters. To prevent false correlation patterns, both covariance estimation and PCA require a large number of independent samples collected from the data. Therefore, the lower the frequencies comprising data are, the longer the sampling time will be for yielding a sufficient number of independent samples.

For deleting slow drifts in the data, we recommend other types of correction methods instead of spatial filtering. Temporal filtering is often more applicable but one must take care not to introduce ringing effects in the data due to the spike artifacts (de Cheveigné and Nelken 2019). In TMS–EEG data, the long decay artifact, thought to represent a depolarization phenomenon in the electrodes, is one special type of slowly changing pattern. Exponential curve fitting has been suggested to remove these long-lasting artifacts (Casula et al. 2017), which can be a good choice if the hypothesis is that these phenomena arise in each channel independently and their time courses obey the exponential decay formula due to depolarization.

### Future Prospects

Here, we validated the usage of the beamforming idea with combined measured and simulated data. We also showed example cases of usage with measured EEG data. We wish to emphasize that there are numerous ways of taking advantage of the presented framework in addition to the presented ideas. Novel measurement techniques and signal-processing steps could improve the estimation of the artifact-spanning topographies in the aim of more accurate cleaning techniques.

More comprehensive artifact elimination could also be performed. Separating the artifacts into groups of correlated components within each group but uncorrelated across the groups can help in designing an efficient preprocessing approach. The groups of artifacts can be set, for example, as each sensor-wise noise signal separately (as in SOUND), ocular artifacts (commonly vertical and horizontal movement topographies), TMS-induced artifacts, peripheral-evoked potentials, and line-noise topographies.

The short computational time required by beamforming for estimating the cleaning matrix can be beneficial for applications where multiple filtering matrices are needed. This happens in the case when the data are non-stationary, i.e, the signals have changing statistics as a function of time. In such a case, the cleaning-matrix estimation should be repeatedly rerun to adapt to the changing statistics. When cleaning TMS-evoked EEG, we can expect that the sensor noise levels are changing as a function of time (Makkonena et al. 2021). Due to the high computationally cost, adaptive cleaning has not been taken into use so far, but the speed of the beamforming computation allows such analysis. In adaptive cleaning, one can update both artifact topographies and the covariance matrix if necessary.

Another time-critical application is real-time EEG cleaning. As spatial filtering requires a linear matrix multiplication with only one EEG sample vector at a time, it is significantly faster than temporal-domain filtering. In Makkonena et al. (2021), SOUND and ICA were used as real-time EEG cleaning algorithms. During an EEG measurement, the data can gradually change, so beamforming is advantageous in efficiently adapting the cleaning operator to the evolving data.

## Appendix: Joint Algorithm for Simultaneously Using SSP and SOUND

To avoid the need to decide the order in which SSP and SOUND were performed, we introduced a modified version, where they are jointly applied as one algorithm as described in the following. The SOUND and SSP–SIR algorithms are thoroughly described in (Mutanen et al. 2018) and (Mutanen et al. 2016), respectively, so here we briefly explain the modifications of the basic ideas.

The idea of SSP is to out-project artifactual topographies from the data, whereas, in SOUND, one channel at a time is interpolated using the rest of the channels. If we wish to exclude the contribution from artifactual data to the interpolation result, we use the SSP out-projection before the interpolation. On the, the out-projection would create artificial spatial correlations over the channels, which is disadvantageous, because the sensor noise must remain uncorrelated for SOUND to work appropriately. As a solution, the out-projection has to be performed separately for each channel during the SOUND iterations such that it will not mix the sensor noise in the channel of interest with the rest of the channels.

To complete the SOUND iteration for channel *j*, we need to construct an out-projection matrix $\textbf{P}^{(j)}$, which is applied to all variables utilized in the process. The interpolation of clean signal in channel *j* is based on the lead-field matrix $\textbf{L}^{(j)}$, the data matrix $\textbf{X}^{(j)}$, and the noise covariance matrix $\Sigma _\textrm{noise}^{(j)}$. The superscript (*j*) denotes the exclusion of the contribution of the *j*th channel from the respective matrix, i.e., the *j*th row in the data and lead-field matrices, and both the *j*th row and column from the covariance matrix are being deleted.

Now, we apply the SSP projection to the data matrix as $\tilde{\textbf{X}}^{(j)}=\textbf{P}^{(j)}\textbf{X}^{(j)}$, to the lead-field matrix as $\tilde{\textbf{L}}^{(j)}=\textbf{P}^{(j)}\textbf{L}^{(j)}$, and to the noise covariance matrix as $\tilde{\Sigma }^{(j)}=\textbf{P}^{(j)}\Sigma _\textrm{noise}^{(j)}\textbf{P}^{(j)\textrm{T}}$. After these operations, the resulting variables, $\tilde{\textbf{X}}^{(j)}$, $\tilde{\textbf{L}}^{(j)}$, and $\tilde{{\Sigma }}^{(j)}$, are used to complete the SOUND iteration for channel *j*. The details are given in (Mutanen et al. 2018). The algorithm then proceeds to the next channel $j'$ to continue with the same process, and the estimation proceeds several times over the channels until convergence.

The projection by SSP is formed as

28 $$\begin{aligned} \textbf{P}^{(j)}=\textbf{I}-\textbf{U}^{(j)}\textbf{U}^{(j)\dagger }, \end{aligned}$$

where $\dagger$ denotes the pseudoinverse operation. In this case, $\textbf{U}$ is first obtained with PCA as usual, such that it would span the artifact subspace. Typically one first applies high-pass-filtering, where after PCA is used to extract the most artifactual components. Then, to transform $\textbf{U}$ into $\textbf{U}^{(j)}$, the *j*th row (channel) is deleted. After the deletion, the principal vectors (columns of $\textbf{U}^{(j)}$) are no longer orthonormal, for which reason projection must be formulated with the help of pseudoinverse instead of the simple transpose in Eq. (28).

Here, we used Laplacian-based high-pass filtering (see (Metsomaa et al. 2021), Appendix A.1) before PCA. With SOUND, the regularization coefficient was set to 0.01.

## Declarations

### Data and Code Availability Statement

The Matlab code for computing beamforming-based cleaning operators is available in an open GitHub repository (https://github.com/jmetsoma/beamforming-based_artifact_removal). The data are not publicly available due to privacy issues. The data used in this study are available from the corresponding author upon reasonable request. A formal data sharing agreement is needed.
